# Large and unequal life expectancy declines during the COVID-19 pandemic in India in 2020

**DOI:** 10.1126/sciadv.adk2070

**Published:** 2024-07-19

**Authors:** Aashish Gupta, Payal Hathi, Murad Banaji, Prankur Gupta, Ridhi Kashyap, Vipul Paikra, Kanika Sharma, Anmol Somanchi, Nikkil Sudharsanan, Sangita Vyas

**Affiliations:** ^1^ Department of Sociology, University of Oxford, 42-43 Park End Street, Oxford OX1 1JD, England.; ^2^ Nuffield College, New Road, Oxford OX1 1NF, England.; ^3^ Leverhulme Centre for Demographic Science, University of Oxford, 42-43 Park End Street, Oxford OX1 1JD, England.; ^4^ Research Institute for Compassionate Economics, 472 Old Colchester Rd., Amston, CT 06231, USA.; ^5^ Department of Demography and Sociology, University of California, Berkeley, 310 Social Sciences Building, Berkeley, CA 94720, USA.; ^6^ Mathematical Institute, University of Oxford, Andrew Wiles Building, Radcliffe Observatory Quarter (550), Woodstock Road, Oxford OX2 6GG, England.; ^7^ Department of Economics, University of Texas at Austin, 2225 Speedway, Austin, TX 78712, USA.; ^8^ Department of Sociology, Emory University, 1555 Dickey Dr, Atlanta, GA 30322, USA.; ^9^ Paris School of Economics, 48 Boulevard Jourdan, 75014 Paris, France.; ^10^ TUM School of Medicine and Health, Technical University of Munich, Georg-Brauchle-Ring 60, 80992 Munich, Germany.; ^11^ Heidelberg Institute of Global Health, Heidelberg University, Im Neuenheimer Feld 130.3, 69120 Heidelberg, Germany.; ^12^ Department of Economics, Hunter College (CUNY), 695 Park Ave., New York, NY 10065, USA.; ^13^ CUNY Institute for Demographic Research, 135 E. 22nd St., New York, NY 10010, USA.

## Abstract

Global population health during the COVID-19 pandemic is poorly understood because of weak mortality monitoring in low- and middle-income countries. High-quality survey data on 765,180 individuals, representative of one-fourth of India’s population, uncover patterns missed by incomplete vital statistics and disease surveillance. Compared to 2019, life expectancy at birth was 2.6 years lower and mortality was 17% higher in 2020, implying 1.19 million excess deaths in 2020. Life expectancy declines in India were larger and had a younger age profile than in high-income countries. Increases in mortality were greater than expected based on observed seroprevalence and international infection fatality rates, most prominently among the youngest and older age groups. In contrast to global patterns, females in India experienced a life expectancy decline that was 1 year larger than losses for males. Marginalized social groups experienced greater declines than the most privileged social group. These findings uncover large and unequal mortality impacts during the pandemic in the world’s most populous country.

## Teaser

High-quality survey data in India find large pandemic mortality impacts on the young, women, and marginalized groups in 2020.

## INTRODUCTION

The COVID-19 pandemic generated a global mortality shock, resulting in large losses in life expectancy worldwide. In high-income countries (HICs), high-quality pandemic surveillance and vital registration systems documented substantial life expectancy declines (*1*, *2*) and increased disparities across race and socioeconomic status (*2*–*6*). However, much remains unknown about the scale and social gradient of COVID-19 mortality in low- and middle-income countries (LMICs), where limited resources imply poor emergency health response, pandemic surveillance, and data quality (*7*–*10*).

This study empirically estimates changes in period life expectancy at birth (hereafter, life expectancy) by sex and social group between 2019 and 2020 in India, where according to the WHO (*11*), one-third of global pandemic excess deaths are estimated to have occurred. Period life expectancy is a summary measure of mortality in a period that enables comparisons of the mortality impacts of the pandemic across populations of different sizes and age structures. We also estimate monthly excess mortality in 2020 relative to baseline. To do this, we use high-quality empirical data on mortality and socioeconomic characteristics from India’s fifth Demographic and Health Survey (DHS), known as the National Family Health Survey-5 (NFHS-5). Using this exceptionally large dataset helps to address major gaps in knowledge about pandemic mortality in India that stem in part from incomplete administrative data and low-quality survey data (*12*–*15*).

**Fig. 1.**
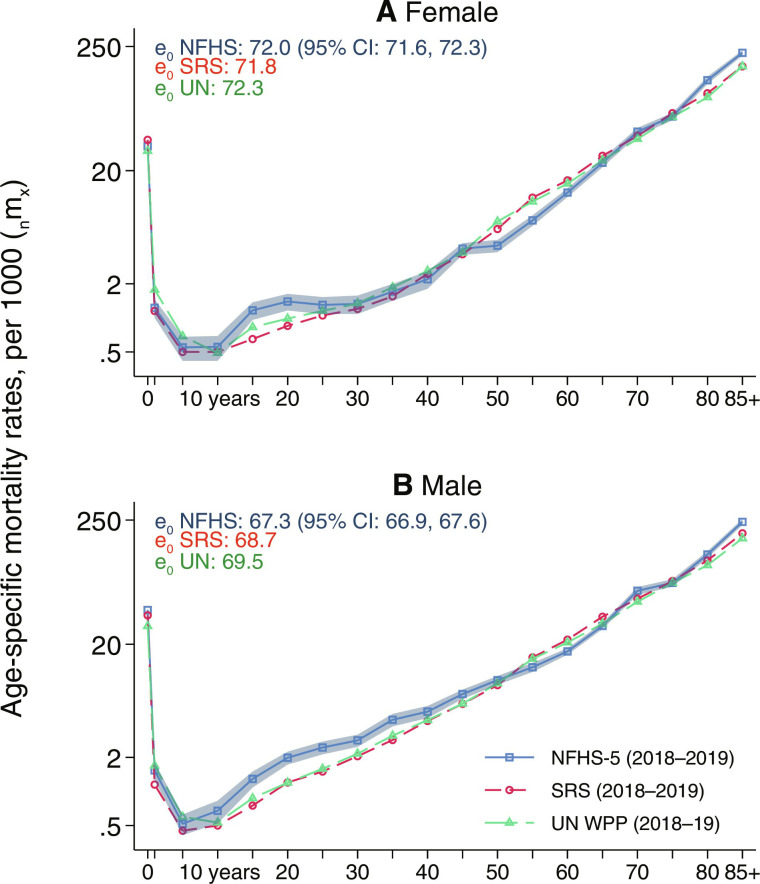
Similar age-specific mortality rates (*_n_m_x_*), from NFHS-5, Sample Registration System (SRS) and UN, 2018 to 2019. National age-specific mortality rates 2018 to 2019 are shown for (**A**) females and (**B**) males, separately, from three different sources. NFHS estimates are for the full NFHS-5 sample (not the subsample). For NFHS-5, mortality rates are estimated on the basis of the procedure described in Materials and Methods, using the full sample. NFHS-5 estimates use sample weights. SRS and UN estimates are mean age-specific mortality rates for 2018 and 2019. 95% CIs for NFHS-5 estimates are shown as the shaded area around estimates and are calculated using a cluster-bootstrap approach. CIs for SRS are not shown because SRS microdata are not publicly available and the reports do not include clustered SEs. CIs are not provided in the UN data. Sources: NFHS-5, SRS 2018 and 2019, and UN WPP 2022.

We first establish the credibility of NFHS mortality rates by comparing them to rates from official sources. Prepandemic mortality estimates from the NFHS closely match life tables from the United Nations (UN) and the government of India’s nationally representative mortality surveillance system [see Fig. 1 and (*16*, *17*)]. Data collection for the fifth round of the NFHS was carried out between 2019 and 2021. We use the subsample of households interviewed in 2021 to study mortality in 2020 relative to prior years. This subsample includes households from 14 states and union territories. It is representative of about one-fourth of India’s population and is similar to the full sample in terms of demographic and socioecomomic characteristics. Unless otherwise noted, all analyses are for the NFHS-5 2021 subsample. In the remainder of this text, we refer to it as the “subsample.”

We find a 2.6-year decline in life expectancy at birth between 2019 and 2020 in the subsample. This decline is larger than the decline in modeled life expectancy estimates in India (*8*, *18*). It is also more than the loss in life expectancy in any HIC in the same period (*1*, *19*). In HICs, life expectancy declines were primarily driven by mortality increases in groups above age 60 (*19*). In India, mortality increased in almost all age groups, most prominently among the youngest and older age groups. It is likely that excess mortality among children was not only due to COVID-19. Among older individuals, excess mortality in 2020 was higher than expected on the basis of age-specific infection fatality rates in HICs and observed seroprevalence in India. Greater observed than expected excess mortality for older age groups could have been due to higher age-specific infection fatality rates in India as well as due to indirect effects of the pandemic.

Our findings demonstrate that the toll of the pandemic was experienced unevenly within India. Whereas in most countries, losses to life expectancy were greater for males than females (*19*, *20*), we document a loss in life expectancy among females that is 1 year more than for males. A larger mortality increase among females relative to males is also evident in India’s vital statistics in subsample states that have high rates of male and female death registration. Among other factors, gender inequality in health care and allocation of resources within households may explain these patterns.

We also find greater life expectancy declines among disadvantaged caste and religious groups relative to privileged social groups in the subsample. Indian society is one of the most stratified in the world. Scheduled Castes (SCs), Scheduled Tribes (STs), and Muslims face social marginalization based on caste, indigenous identity, and religion, respectively (*21*–*23*). Relative to a decline in life expectancy of 1.3 years for high-caste Hindus, who are privileged in Indian society, the loss for Muslims was 5.4 years, for STs was 4.1 years, and for SCs was 2.7 years. Before the pandemic began, each of these three groups faced large disadvantages in life expectancy at birth relative to high-caste Hindus (*17*, *24*). The pandemic exacerbated these disparities. These declines are comparable or larger in absolute magnitude to those experienced by Native Americans, Blacks, and Hispanics in the United States in 2020 (*3*, *25*).

We find that mortality in the subsample was 17.1% higher in the pandemic months of 2020 relative to 2019 and was particularly elevated in the last 4 months of 2020. These estimates are robust to alternative baselines. While likely smaller than the 2021 mortality surge (*26*), our results show that if the rest of India also experienced an increase in 2020 mortality similar to the subsample, it would imply 1.19 million excess deaths in 2020 nationally. Relative to other estimates during the same period, our extrapolated estimate for all-India excess deaths is about eight times the official number of COVID-19 deaths in India (*27*), 1.5 times the WHO’s extrapolated estimate of excess deaths in India (*28*), and more than 2.5 times the estimated excess deaths in the United States in 2020 (*29*).

Our estimates using high-quality NFHS data fill an important gap in scientific understanding of pandemic mortality in India and globally in 2020. Administrative data from India’s Civil Registration System (CRS), which have been used for many existing estimates of excess mortality in India (*26*, *30*–*32*), including the WHO estimates (*11*, *28*), do not capture all births and deaths, are unavailable for many states, and were disrupted by India’s severe lockdown in 2020. Although informative for highlighting weaknesses of administrative data, existing surveys that have been used for prior research on India’s pandemic mortality are not representative (*33*) and do not produce accurate estimates of baseline mortality (*31*, *34*).

Because India is the most populous country in the world, understanding the global toll of the pandemic relies on accurately estimating pandemic mortality in India. Our findings uncover large and unequal effects of the pandemic in India and show that disadvantages can be exacerbated in times of a mortality crisis. Methodologically, our analysis demonstrates the value of empirical approaches using high-quality data to measure routine and crisis mortality. In particular, our analysis underscores that large-scale sample surveys that ask questions on recent deaths of household members are valuable for mortality surveillance in data sparse settings. These approaches may reveal empirical patterns missed by modeling approaches or nonrepresentative and low-quality data sources.

## RESULTS

### Increase in mortality between 2019 and 2020 by age and sex

**Fig. 2.**
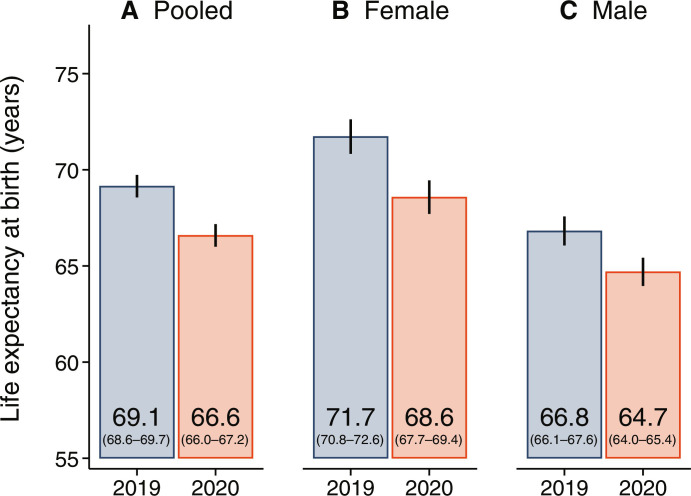
Declines in period life expectancy between 2019 and 2020 are large and patterned by gender. The figure shows life expectancy at birth in 2019 and 2020 for the (**A**) combined female and male population, (**B**) females, and (**C**) males, separately. Life expectancy is calculated on the basis of standard life table procedures. Estimates are for the NFHS-5 2021 subsample and use sample weights. The vertical lines around each estimate represent 95% CIs calculated using a cluster-bootstrap approach. See fig. S1 for estimates of the decline in life expectancy between the 2 years. Source: NFHS-5.

Figure 2 shows estimates of life expectancy at birth in 2019 and 2020 for females, males, and the combined population; fig. S1 shows estimates of the change between the two periods. The vertical lines represent 95% confidence intervals (CIs). Between 2019 and 2020, overall life expectancy at birth declined by 2.6 years (95% CI: 1.8 to 3.3) in the NFHS-5 2021 subsample. Figure S2 shows that this decline was similar in rural and urban areas. This drop is greater than the decline in life expectancy observed in the same period in any HIC (*1*). It is also larger than the reduction between 2019 and 2020 for India modeled by the UN (*18*) and others (*8*). Discrepancies across studies may in part be due to variation in the quality of the underlying data or to different mortality patterns in non-subsample regions. However, in order for NFHS-5 subsample estimates to be consistent with modeled estimates (*8*, *18*), life expectancy in non-subsample regions would have had to decline by as little as 0.1 to 0.4 years, which is an unlikely scenario.

This overall decline masks substantial heterogeneity across gender. Increases in mortality over this period narrowed the life expectancy advantage observed among females in the subsample. Relative to a decline of 2.1 years (95% CI: 1.1 to 3.2) observed among males, the decline for females was 1 year larger at 3.1 years (95% CI: 1.9 to 4.4). The 95% CI for the difference in decline between females and males is −0.6 to 2.6 years. Although we cannot rule out that the greater decline for females relative to males is due to sampling error, greater declines for females relative to males are also observed in vital statistics. Civil registration data in NFHS-5 subsample states that had high rates of prepandemic death registration for females and males (see table S1 for the states that meet this criteria) also showed larger increases in mortality between 2019 and 2020 for females compared to males in most states, as shown in fig. S3. These patterns are in stark contrast to the global pattern of a greater increase in mortality during the COVID-19 pandemic for males compared to females (*1*, *20*, *35*). Such a large female disadvantage in the impact of the pandemic as observed in India has not been documented in any country.

Greater declines for females relative to males in India may reflect gender inequality. Gender inequality in India has long been shown to reduce the female advantage that we would expect in the absence of discrimination against girls and women (*36*, *37*). Consistent with gender inequality as an explanation, we observed larger losses among females compared to males among all social groups except for STs, among whom gender disparities are lower (*22*, *38*). Prior research has documented that Indian households spend less on health care for females relative to males (*39*, *40*), a pattern which likely worsened during the pandemic (*41*, *42*). Gender inequality is also suggested by the underrepresentation of females in India’s official COVID-19 case data (*15*) despite similar levels of seroprevalence relative to males in sample surveys (*43*).

**Fig. 3.**
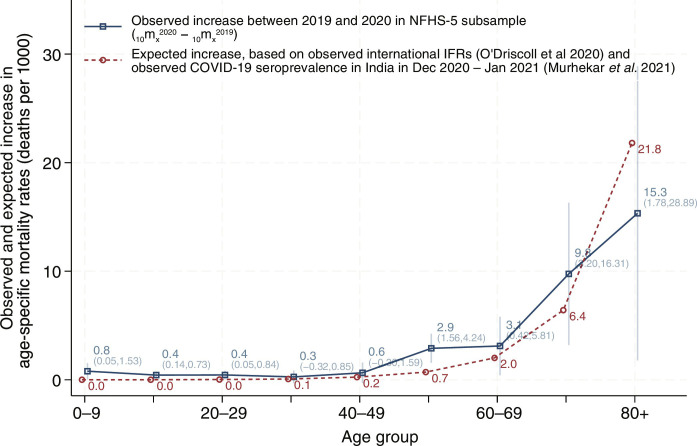
Comparison of observed and expected increase in age-specific mortality rates. The figure shows the observed and expected increase in age-specific mortality rates between 2019 and 2020. The observed increase in age-specific mortality is estimated on the basis of the NFHS-5 2021 subsample, comparing age-specific mortality rates between 2019 and 2020. We report the absolute increase in mortality in 10-year age intervals. The expected increase is calculated as a product of observed spread of COVID-19 in India (from a serosurvey) and observed infection fatality rates (IFRs) observed in HICs. The third national serosurvey, conducted in December 2020 and January 2021, found seroprevalence to be 24.1% (*43*). Although the serosurvey shows similar spread across ages, we use age-specific seroprevalence to calculate the expected increase in mortality. IFRs are based on seroprevalence and death records from HICs. Source: NFHS-5, (*43*), and (*44*).

Figure 3 shows the absolute increase in age-specific mortality rates between 2019 and 2020 and compares this to the expected increase in mortality based on observed infection fatality rates in HICs from O’Driscoll *et al.* (*44*) and observed COVID-19 seroprevalence in India in December 2020 to January 2021 from Murhekar *et al.* (*43*). 95% CIs around observed excess mortality are indicated by vertical lines. The figure shows that observed excess mortality was higher than the expected increase across the life course but particularly in childhood and at ages 50 to 60. Excess mortality in the youngest ages is consistent with increased vulnerability of children to COVID-19 in contexts with comparatively higher deficits in early-life health (*45*) and existing evidence on indirect effects of the pandemic and lockdown (*42*, *46*, *47*). Prior research has identified deteriorating economic conditions (*48*) and pandemic disruptions to public health services such as childhood immunizations (*49*), tuberculosis treatment (*50*), and hospital births (*51*). These disruptions might have been partly responsible for an increase in all-cause mortality in 2020. Among older age groups, higher observed compared to expected mortality could have been due to higher age-specific infection fatality rates in India compared to HICs, as hypothesized by Nepomuceno *et al.* 2020 (*10*) and Levin *et al.* 2022 (*52*), or indirect effects of the pandemic.

**Fig. 4.**
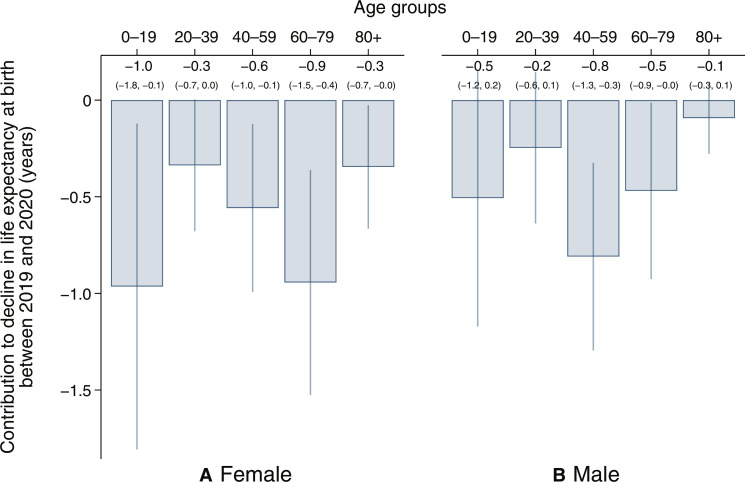
Increases in mortality in younger age groups contributed more than older age groups to declines in period life expectancy at birth between 2019 and 2020. The figure shows the decomposition of the change in life expectancy between 2019 and 2020 into contributions from changes in mortality in different age groups based on Arriaga’s decomposition for (**A**) females and (**B**) males (*79*). Estimates are for the NFHS-5 2021 subsample and use sample weights. 95% CIs are shown as vertical lines around estimates and are calculated using a cluster-bootstrap approach. Source: NFHS-5.

Whereas Fig. 3 helps understand age-specific contributions to changes in all-cause mortality, we use an Arriaga decomposition to understand age-specific contributions to changes in life expectancy. Figure 4 shows the absolute contribution, in years, of increases in mortality in different age groups to declines in life expectancy at birth between 2019 and 2020. The panel on the left shows results for females, and the panel on the right shows results for males. 95% CIs are indicated by vertical lines. Although absolute increases in mortality were highest in the oldest ages (from Fig. 3), mortality increases in the younger ages contributed substantially to life expectancy losses because life expectancy is more sensitive to early-life mortality. For females, mortality increases in the 0 to 19 and 60 to 79 ages contributed the most, approximately 1 year each (95% CIs: −1.8 to −0.1 and −1.5 to −0.4, respectively), to the decline in life expectancy. Figure S4, which shows age-specific contributions to life expectancy declines by abridged life table age groups, reveals that the declines for females were particularly large during childhood, the early reproductive years, and above age 60. For males, mortality increases in the 40 to 59 ages contributed the most, 0.8 years (95% CI: −1.3 to −0.3), to the decline. These findings contrast sharply with age-specific contributions to changes in life expectancy in HICs, where most of the decline in life expectancy in 2020 was contributed by mortality increases above age 60 and especially above age 80 (*35*).

### Greater life expectancy declines among marginalized social groups

**Fig. 5.**
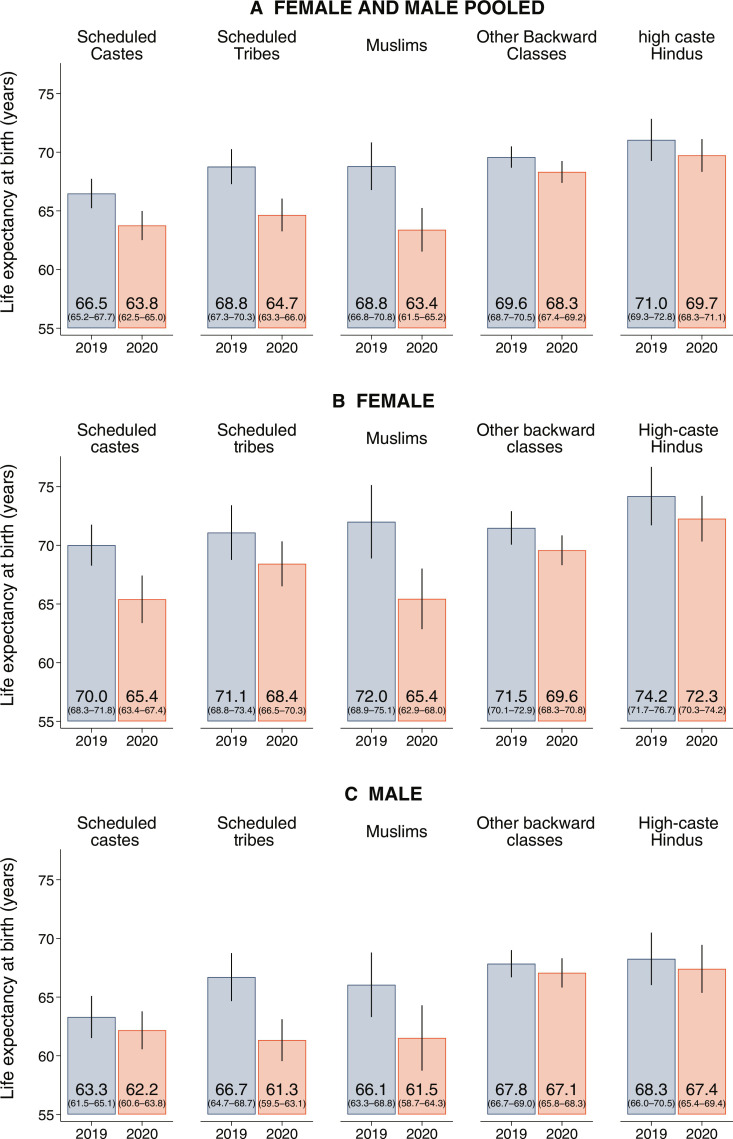
Greater declines in period life expectancy between 2019 and 2020 for disadvantaged social groups compared to privileged social groups. The figure shows life expectancy at birth in 2019 and 2020 for the (**A**) combined female and male population, (**B**) females, and (**C**) males, separately. Life expectancy is calculated on the basis of standard life table procedures. Estimates are for the NFHS-5 2021 subsample and use sample weights. The vertical lines around each estimate represent 95% CIs calculated using a cluster-bootstrap approach. See fig. S1 for estimates of the decline in life expectancy between the 2 years. Source: NFHS-5.

India is a highly unequal society. For each social group separately, Fig. 5 shows estimates of life expectancy at birth in 2019 and 2020, and fig. S1 shows estimates of the change between the 2 years. Figure 5A shows estimates for the combined population, Fig. 5 [(B) for females and (C) for males]. Estimates for SCs are shown in the first column, STs in the second column, Muslims in the third column, Other Backward Classes (OBCs) in the fourth column, and high-caste Hindus in the last column. Vertical lines represent 95% CI.

Marginalized social groups experienced the largest reductions in life expectancy at birth, which exacerbated already existing social inequalities. Relative to a 1.3-year (95% CI: −1.0 to 3.6) life expectancy loss among high-caste Hindus, the loss for Muslims was 5.4 years (95% CI: 2.8 to 8.1), for STs was 4.1 years (95% CI: 2.0 to 6.2), and for SCs was 2.7 years (95% CI: 1.0 to 4.5). The decline for OBCs of 1.3 years (95% CI: −0.1 to 2.6) was similar in magnitude to that among high-caste Hindus. The 95% CI for the difference in decline between Muslims and high-caste Hindus was 0.7 to 7.5 years, between SCs and high-caste Hindus was −1.5 to 4.3 years, and between STs and high-caste Hindus was −0.2 to 5.8 years.

In 2020, Muslim life expectancy was the lowest across the five social groups, a result of the fact that Muslims observed the greatest declines in life expectancy at birth between 2019 and 2020. This decline is consistent with the further marginalization of Muslims in 2020 (*53*) and with a greater increase in neonatal mortality among Muslims (*46*).

These declines among marginalized groups are similar or greater in absolute magnitude than declines experienced by Blacks, Hispanics, and Native Americans in the United States during the pandemic (*2*, *25*, *54*). They are larger in relative magnitude, although since Indian subgroups had lower baseline life expectancy levels in 2019. In the United States, life expectancy at birth declined by 3.3 years from its 2019 level of 74.8 years among Blacks, 4.0 years from its 2019 level of 81.9 years among Hispanics, and 4.5 years from its 2019 level of 71.8 years among Native Americans (*25*, *55*, *56*).

### Excess mortality in 2020

**Fig. 6.**
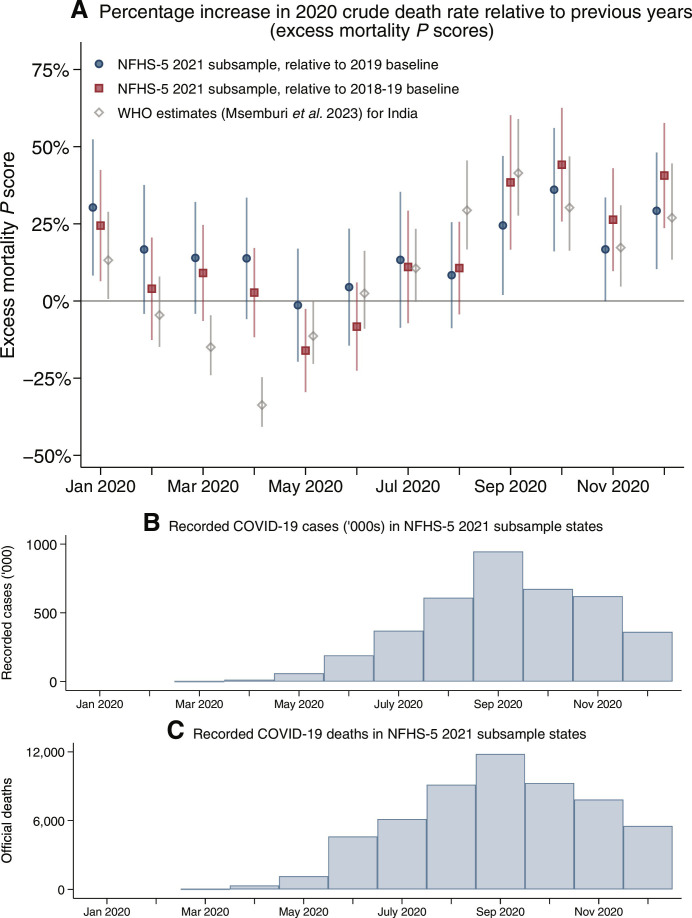
Greater percentage increase in mortality in the last 4 months of 2020 relative to prior years. (**A**) shows excess mortality estimated in the NFHS-5 2021 subsample and compares it to excess mortality estimated by the WHO (*11*). For the NFHS-5 2021 subsample, monthly excess mortality *P* scores are estimated on the basis of the procedure described in Materials and Methods. The percentage increase in monthly crude death rates in 2020 is shown relative to two different baselines: 2019 only and the average of mortality in 2018 and 2019. NFHS-5 estimates use sample weights, and 95% CIs calculated using a cluster-bootstrap approach are shown as vertical lines around estimates. WHO estimates are extrapolated for India based on data from 17 states and union territories. 95% CIs for WHO estimates are also shown as vertical lines and were provided in (*11*). (**B**) COVID-19 cases and (**C**) COVID-19 deaths, reported by laboratories and hospitals to state agencies. Sources: NFHS-5, (*11*), and (*27*).

Figure 6 displays monthly estimates of 2020 excess mortality from the NFHS-5 2021 subsample and compares these results to WHO estimates of excess mortality (*11*) and official records of case counts and deaths (*27*). We compute excess mortality using two different baseline mortality estimates: the same calendar month in 2019 and the average over the same calendar month in 2018 and 2019. Vertical lines represent 95% CIs.

Mortality was elevated from September to December 2020, relative to the same months in the previous years (Fig. 6A). On average between April and December 2020, mortality was 17.1% (95% CI: 10.5 to 23.7) higher than in 2019. Excess mortality was higher for females than males. Table S2 estimates excess deaths nationally based on these findings. If excess mortality from the NFHS-5 2021 subsample were observed nationally, it would imply 1.19 million (95% CI: 0.73 to 1.65) excess deaths in 2020.

We verify that these results are robust in several ways (*57*, *58*). First, using mean mortality in 2018 and 2019 as an alternative baseline, the 2020 excess mortality *P* score is 16.9% (95% CI: 11.3 to 22.6) in the pandemic months. Second, to ensure that these results are not driven by the particular states that are in the subsample, we calculate excess mortality *P* scores by constructing hypothetical sub-subsamples (subsamples of our NFHS-5 2021 subsample), which exclude one state at a time (*11*, *26*). Figure S5 shows that excess mortality is similar across hypothetical sub-subsamples. Between the NFHS-5 2021 subsample and the full NFHS-5 sample, however, there are differences in the proportion that is Hindu, Muslim, ST, OBC, and Other caste. These compositional differences might contribute to differential excess mortality rates between subsample and non-subsample places.

Figure 6A also compares NFHS-5 subsample excess mortality to WHO estimates, which use incomplete CRS data from 17 states and union territories and extrapolate to the rest of the country. Both sources show elevated mortality from September through December 2020. However, NFHS-5 estimates do not display negative excess mortality in March, April, and May 2020, which the WHO estimates suggest. The reduction in mortality in the WHO data was likely due to a disruption of vital registration during the strict lockdown during these months (*12*). Disruption in registration during India’s lockdown would not have affected mortality estimates from the NFHS-5 because they are based on respondents reporting retrospectively in a survey on deaths that occurred in their household.

Figure 6 (B and C) displays monthly COVID-19 cases and deaths reported by laboratories and hospitals to state agencies. These data were compiled by COVID19India.org (*27*). Figure 6 (B and C) shows a peak in cases and deaths in September 2020 and a subsequent decline. This pattern is inconsistent with the NFHS-5 subsample and WHO estimates, which both show elevated mortality from September through December 2020. One reason for the discrepancy across data sources could arise from an increase in mortality from causes other than COVID-19 in the latter part of the year, which would show up in NFHS-5 and WHO estimates but not pandemic surveillance. Another reason could be uneven pandemic surveillance (*59*). Figure S6, which shows NFHS-5 estimates of annualized age-standardized crude death rates by month for urban and rural areas separately, suggests that official surveillance may have more closely tracked COVID-19 mortality in urban areas and missed mortality dynamics in rural areas. Rural seropositivity increased substantially from 5.6 to 21.4% between August to September 2020 (*60*) and December 2020 to January 2021 (*43*). Both the NFHS-5 subsample and serosurvey data provide evidence that COVID-19 was spreading in rural areas between September and December 2020 even as recorded cases and official COVID-19 deaths from a flawed disease surveillance system were declining.

## DISCUSSION

This study uses high-quality empirical data to understand the scale, distribution, and disparities in excess mortality during the first year of the pandemic in India, where a substantial fraction of global pandemic deaths are estimated to have occurred but where impacts have been uncertain. In our subsample, which represents one-fourth of India’s population, we find a reduction in life expectancy at birth of 2.6 years between 2019 and 2020, larger than reductions documented in any HIC. This reduction was substantial, even relative, to trends in India: Overall life expectancy at birth in 2020 was equivalent to all-India levels over a decade earlier (*61*). Our estimates imply 1.19 million excess deaths in India nationally in 2020, which is about one-third of the excess deaths estimated by the WHO for the rest of the world (*11*). From a comparative perspective, India’s life expectancy decline is similar to or larger than declines seen in the same period in other large LMICs including Brazil (*62*, *63*), Russia (*35*), and Mexico (*64*). Excess mortality *P* scores in India are similar to those observed in Eastern Europe (*65*) and lower than those observed in LMICs such as Ecuador (*4*). However, excess mortality in India displays a younger age profile.

The increase in mortality between 2019 and 2020 was heterogeneous across age, sex, and social group. Although mortality increased across all ages in India, relative to HICs, increases in mortality in younger age groups (under age 60) contributed more to life expectancy declines. In contrast to global patterns, we find that life expectancy declined by one full year more for females than for males. Like in the United States, where the pandemic increased existing gaps in life expectancy, such as those by race, ethnicity, and education (*2*, *5*, *54*), gaps in India between privileged and marginalized groups also increased. Relative to high-caste Hindus, the gap in life expectancy at birth for SCs increased from 4.5 years in 2019 to 5.9 years in 2020; for STs, it increased from 2.2 to 5.0 years, and for Muslims, it increased from 2.2 to 6.3 years.

The NFHS-5 is a rich data source that uncovers patterns of mortality that could not be observed in other data sources. India’s CRS, which records vital events, is incomplete even in normal times with lower coverage among females, marginalized groups, and the young (*66*). It was also disrupted during India’s severe lockdown in March through May of 2020 (*26*). These factors may explain why the WHO estimates, which are based on CRS data from 17 states and union territories, are biased downward. Similarly, recorded case and death counts in India missed most infections and COVID-19 deaths (*26*, *43*, *67*). They are also more likely to capture males than females (*15*, *68*) due to gender inequality in access to health care (*39*). In Kerala, perhaps the only state where mortality surveillance was robust, COVID-19 mortality was higher among female children relative to male children (*13*). Yet, Kerala is unique in that gender inequality is less severe and COVID-19 spread in 2020 was low. We additionally find evidence that recorded case and death counts more closely tracked urban than rural excess mortality. These data would have also missed mortality patterns from causes other than COVID-19. Our findings highlight the inherent risks in inferring mortality patterns from recorded COVID-19 case and death data. Last, existing survey-based estimates of excess mortality are based on unrepresentative samples, which underrepresent women, young children, rural areas, and the poor (*33*).

Our findings have important implications for further research. For example, more clarity is needed to understand why females in India fared worse than males in terms of life expectancy losses and excess mortality, why the age profile of excess mortality was younger in India than in other countries, and why Muslims suffered such high losses to life expectancy at birth relative to other social groups. Age and gender patterns showing that females and younger age groups in India have been disproportionately affected by the pandemic suggest that indirect effects of the pandemic and lockdown may have contributed to the overall increase in mortality. However, additional data are needed to understand direct versus indirect mortality impacts of the COVID-19 pandemic in 2020 and beyond.

Another important remaining gap in our understanding relates to changes in mortality in regions that were not represented in the NFHS-5 2021 subsample. Although our 2021 subsample is similar to the full NFHS-5 sample on many characteristics, the subsample has a different social and religious composition compared to the full sample. In addition, subsample interviews are geographically clustered in 14 states and union territories. For these reasons, our results from analyses of the subsample may not provide a full understanding of changes in mortality at the national level in 2020 compared to 2019. However, evidence suggests that changes in mortality in this subsample might not have been so different from regions that are not in this subsample. India’s third national serosurvey (*43*) shows that disease spread was similar in the subsample states compared to states that were not in the subsample (see table S3). In addition, our estimates of excess mortality are not driven by any one state and are similar in hypothetical sub-subsamples (subsamples of the NFHS-5 2021 subsample), which exclude one state at a time (see fig. S5).

Methodologically, our study shows the potential for accurately estimating mortality, even for shorter periods, using retrospective mortality information collected in a large-scale sample survey in a relatively poor context. These approaches may be helpful in routine mortality surveillance and understanding other mortality crises using in-person or phone surveys (*69*). The extent to which retrospective questions on deaths of household members estimate mortality well in other contexts is a topic for further research. In particular, biases arising from recall errors, noncoverage of households in which all members died, and household dissolution may be more important in other contexts (*16*, *24*, *70*).

From a policy perspective, it is clear that the pandemic exacerbated longstanding inequalities in population health, particularly along dimensions of caste, religion, indigenous identity, rural or urban residence, age, and sex. In showing that pandemics can exacerbate inequalities rather than level them (*71*, *72*), our findings reinforce the relevance of perspectives that emphasize social conditions as a fundamental cause of health and mortality (*73*). Although pandemic mortality in India in 2020 did not receive the same attention as the 2021 surge due to the Delta variant, our results show large and unequal mortality increases even early in the pandemic. Overall, the findings suggest that a greater focus on disadvantaged groups such as females, marginalized populations, and rural areas is important in understanding and responding to future mortality crises.

## MATERIALS AND METHODS

### The main sample: NFHS-5 subsample of households interviewed in 2021

This study uses data from the NFHS-5, which is India’s DHS. In particular, we use the subsample of households interviewed in 2021 to study mortality in 2020 relative to prior years. We estimate mortality for 2018, 2019, and 2020 all from the same households interviewed in 2021 using retrospective questions on mortality. Results are therefore not biased by comparing mortality pre- and post-pandemic between groups that differ on characteristics.

Data collection for the NFHS-5 was scheduled over two phases. Phase1 states were interviewed in 2019 and the first 2 months of 2020. Interviews in phase 2 states began in late 2019 but were disrupted by the severe lockdown in March 2020. Households interviewed in phase 1 states and in phase 2 states before the lockdown in March 2020 are not part of the main analysis sample because they were interviewed before the pandemic began. Data collection resumed in phase 2 states in October 2020 and continued until May 2021 when the surge in mortality due to the Delta wave prevented further interviews. As fig. S7 shows, the phase 2 households interviewed in 2021 comprise the main analysis sample for this study. We do not include the phase 2 state households interviewed in the last quarter of 2020 because we estimate mortality for these months. Our subsample of households interviewed in 2021 is representative of 23.2% of the Indian population.

Evidence suggests that data quality between phase 1 and phase 2 states is similar. The response rate was 98% in phase 1 states and 97% in phase 2 states (*74*). The small difference in response rate between the two sets of states suggests that there was not a dramatic increase in refusals in 2021. The NFHS-5 report also makes note of survey procedures undertaken to maintain data quality during the pandemic [pg. 11, (*74*)].

Figure S8 shows the proportion of interviews conducted in each calendar month for each of the 36 states and union territories where NFHS-5 was fielded, and fig. S9 displays a map of primary sampling units (PSUs) that are and are not part of the main analysis sample. Of 36 states, interviews were conducted in early 2021 in 14 states and union territories: Punjab, Chandigarh, Uttarakhand, Haryana, Delhi, Rajasthan, Uttar Pradesh, Arunachal Pradesh, Jharkhand, Odisha, Chhattisgarh, Madhya Pradesh, Tamil Nadu, and Puducherry.

### Other data

We use several other data sources for comparative purposes and computations:

• Age-specific mortality rates from the Sample Registration System (SRS), which is the government of India’s nationally representative mortality surveillance system (*61*). The SRS collects data on all vital events in sample villages. This sample surveillance system is distinct from India’s CRS, which is meant to record all vital events nationally but is still incomplete because many births and deaths are not registered (*12*, *66*).

• State- and sex-specific deaths recorded by India’s CRS (*75*) for the years 2019 and 2020.

• WHO estimates of excess mortality from Msemburi *et al.* 2023 (*11*). Estimates for India by the WHO were constructed on the basis of data from India’s CRS, which, in addition to being incomplete, are only available for a portion of the pandemic period for 17 states and union territories. In addition, the CRS was disrupted by India’s severe lockdown in 2020.

• Recorded COVID-19 cases and COVID-19 deaths, which were reported by laboratories and hospitals to state agencies and compiled by covid19india.org (*27*).

• Seroprevalence from Murhekar *et al.* (*43*), a nationally representative serosurvey carried out in December 2020 to January 2021.

• Estimated age-specific mortality rates, deaths, and age distribution of the Indian population from UN World Population Prospects (WPP) (*18*).

### Estimating age-specific mortality rates and life expectancy at birth

We follow the approach described in Gupta (*16*) and Gupta and Sudharsanan (*17*) to estimate age-specific mortality rates from the NFHS. We use standard demographic approaches (*76*, *77*) to estimate mortality under age 2 from the birth history module of NFHS-5. For mortality at age two and above, we use questions on deaths in the household since January 2017 to estimate deaths and person-years lived by those who did not survive. In addition to the age and sex of each deceased household member, information on the month and year of death was also collected. For those who were alive at the time of the interview, we use the household roster, which contains the age of each individual in the household, to estimate person-years lived. Table S4 shows the number of deaths and person-years in each year, by age, sex, and social group.

We estimate age-specific mortality rates by creating empirical lifelines at the calendar-month level for those who died and for those who were alive at the time of the survey. Age-, sex-, month-, and social group–specific mortality rates are estimated separately using the formula

$${\;}_{n}m_{x}^{t,s,g}=\frac{\sum_{i=1}^{I}‍{\left(\text{died between}\;\mathrm{age}\;x\;\text{to}\;x+n\;\text{in}\;\mathrm{mon}th\;t\right)}_{i}\times \left(\text{weigh}t_{i}\right)}{\sum_{i=1}^{I}‍{\left(\text{person}\;\text{months}\;\text{lived}\;\text{between}\;\mathrm{age}\;x\;\text{to}\;x+n\;\text{in}\;\text{month}\;t\right)}_{i}\times \left(\text{weigh}t_{i}\right)}$$ (1)

where *m* refers to mortality rate, *x* to the interval’s beginning age, *n* to the length of the age interval, *t* to month, *s* to sex, and *g* to social group. We use the abridged life table age ranges used by the SRS: 0 to 1, 1 to 5, 5 to 10, ..., 80 to 85, and 85+. *i* refers to individuals and *I* to the total number of individuals of sex *s* and group *g* covered in the survey. Observations use sample weights and are representative of the NFHS-5 2021 subsample. Figure S10 shows age-specific mortality rates estimated using these procedures in 2019 and 2020 separately.

Using standard approaches (*78*), we construct period life tables to calculate life expectancy at birth in 2019 and 2020 separately. We use the following equation to estimate life expectancy:

$$e_{x}=\frac{\int_{x}^{\infty }‍l\left(a\right)\mathrm{da}}{l\left(x\right)}$$ (2)

where *x* is the age for which life expectancy is calculated, $\int_{x}^{\infty }‍l\left(a\right)\mathrm{da}$ represents person-years lived above age *x*, and *l*(*x*) stands for survivors to age *x*. In discrete terms, this corresponds to *T_x_*/*l_x_*, where *T_x_*, total person-years lived above age *x*, is calculated as the sum of person-years lived by a hypothetical cohort, which experiences the set of age-specific mortality rates in each age interval after age *x*. The average number of person-years lived by those who died in an age interval, or *_n_a_x_*, is borrowed from 2015 to 2019 official SRS life tables.

We first compare life expectancy at birth in 2019 to 2020 for the full subsample and separately for females and males. To explore disparities in life expectancy changes, we estimate life expectancy at birth in 2019 and 2020 by social group, following the same social categories as Gupta and Sudharsanan (*17*). We compare high-caste Hindus, who are relatively privileged in Indian society, to four marginalized social groups: SCs, STs, Muslims, and OBCs.

To assess the importance of changes in mortality in different age groups to the overall change in life expectancy at birth between 2019 and 2020, we apply Arriaga’s decomposition (*79*). We estimate the contribution of differences in mortality between ages 0 and 19, 20 and 39, 40 and 59, 60 and 79, and 80+ to the change in life expectancy between 2019 and 2020 for females and males separately.

### Estimating excess mortality

To study excess mortality in 2020 relative to prior years, we estimate crude death rates for each calendar month between January 2018 and December 2020. We annualize these estimates of monthly death rates to enable comparisons across periods that vary in length. Annualized monthly death rates can be interpreted as expected mortality in a year if the mortality in the month under consideration was observed for an entire year. Crude death rates have also been age-standardized using the 2020 population age distribution, estimated by the UN WPP (*18*).

Figure S6 shows crude death rates calculated using this approach for each calendar month in 2018, 2019, and 2020. For each month in 2020, we use these crude death rates to calculate the excess mortality *P* score, which is the percentage increase in mortality in a particular month relative to that same month in the baseline period (*26*). As baseline mortality, we consider mortality observed in 2019 and average mortality observed in 2018 and 2019. Both options produce similar results for excess mortality. These estimates may actually be conservative because mortality in 2020 would likely have been slightly lower than in prior years in the absence of a pandemic. Excess mortality *P* scores can be calculated as follows:

$$P-\text{scor}e^{m}=\frac{\text{observed}\;\text{death}\;\mathrm{rat}e^{m,2020}-\text{observed}\;\text{death}\;\mathrm{rat}e^{m,\text{baseline}}}{\text{observed}\;\text{death}\;\mathrm{rat}e^{m,\text{baseline}}}\times 100$$ (3)

We calculate *P* scores for each calendar month *m* in 2020 separately. We also calculate total excess mortality in April 2020 to December 2020 (table S2). For this, we consider mortality in April to December 2019 as the baseline.

All analyses use survey weights. To calculate 95% CIs, we use a cluster-bootstrap approach that replicates the multistage sampling structure of NFHS-5 and accounts for clustering of observations within PSUs (*80*).

### Robustness checks

#### Representativeness of the NFHS-5 2021 subsample

Tables S5 to S8 compare the 2021 subsample to the full NFHS-5 sample. The subsample is similar to the full sample in terms of distribution by urban and rural residence, sex, and the proportion of the population that identifies as SC. There are differences between the subsample and the full sample in the proportion of the population that is Hindu, Muslim, ST, and OBC. Prior studies examining social disadvantage in mortality have found that STs have the lowest life expectancy in India (*17*, *24*). Perhaps because of the regional composition of social groups in the subsample, we found that SCs had the lowest life expectancy among all social groups in 2019. The subsample is also slightly younger than the full sample, with a greater proportion of the population that is below age five.

These compositional differences have implications for the national-level estimate of excess mortality for 2020. Our results show that increases in mortality between 2019 and 2020 varied by age and social group. Differences between the subsample and the full sample on these dimensions, therefore, reduce confidence in national-level extrapolations. The relative status of social groups, however, also differs across regions (*17*), which complicates reweighting the subsample to match the national-level demographic and social composition of the population. For simplicity, our national-level excess death estimate does not account for compositional differences between the NFHS-5 2021 subsample and the full sample.

On other indicators of economic status, differences are relatively minor and not systematically in favor of the subsample or the full sample. For example, in the subsample, household sizes are slightly larger, a greater proportion own a two-wheeler, but television ownership is lower. The subsample and the full sample are similar in terms of access to toilets, but access to piped water and clean fuels is lower in the subsample. Children are slightly taller and heavier in the subsample.

On mortality, fig. S11 shows that households in the subsample (the 2021 interviews) did not have substantially different mortality rates compared to households interviewed in 2019 or 2020. Figures 1 and 2 also show that estimates of life expectancy at birth in the prepandemic period were similar between the 2021 subsample and the full sample. Among women, life expectancy at birth was 71.7 years in the subsample in 2019 and 72.0 years in the full sample in 2018 to 2019. In the same years among men, life expectancy at birth was 66.8 years in the subsample and 67.3 years in the full sample. These similarities provide evidence that subsample areas had similar levels of mortality to the rest of the country before the pandemic.

Although the 2021 subsample is geographically clustered and the spread of the pandemic may have varied spatially, table S3 shows that disease spread at the end of 2020, as measured in the third national serosurvey (*43*), was similar in states visited by the NFHS-5 in 2021 compared to states visited earlier. We also show that our results are not driven by any single state and are robust to excluding one state at a time in hypothetical sub-subsamples (subsamples of our NFHS-5 2021 subsample) in fig. S5.

#### Reliability of NFHS-5 mortality rates

Figure 1 shows that age-specific mortality rates for 2018 to 2019 computed from the full NFHS-5 sample (not the subsample) are similar to those in the same period from the UN WPP and the SRS. 95% CIs for NFHS-5 rates are shown as the shaded area around the NFHS estimates. SRS and UN do not provide CIs for age-specific mortality rates. For some age groups, particularly in young adulthood when mortality is low, NFHS-5 mortality rates are higher than those in the SRS. These differences contribute to relatively minor differences in life expectancy at birth between the NFHS and SRS. Differences between data sources are slightly larger for males than females, but both are in line with differences between data sources observed in prior research in India (*17*) and elsewhere (*81*, *82*).

In addition to the fact that SRS microdata are not made publicly available, SRS reports do not describe the methods used for constructing age-specific mortality rates. In the past, NFHS and SRS age-specific mortality rates were more similar, with both showing a mortality hump in young adulthood (*17*). However, even before the pandemic, several inconsistencies in the SRS had been described, such as a crude death rate that was lower than the UN WPP, a younger age structure than the Indian Census and the UN WPP, and lower infant mortality in poorer states such as Bihar than estimated by the NFHS (*12*). Franz *et al.* (*83*) also demonstrated that the SRS underestimated maternal mortality relative to the NFHS. Without more information on SRS mortality estimation procedures, it is not possible to reject the possibility that the SRS actually underestimates mortality in young adulthood in 2018 to 2019.

A final concern is that mortality in households in which all members have died is missed using our methods. This is more likely to occur among more disadvantaged households and would have been more likely in 2020 than in 2019. This type of bias implies that our findings represent a conservative estimate of gaps between social groups and declines between baseline and pandemic period.

#### Little evidence of recall bias

Recall bias is a concern when using retrospective mortality data from household surveys and, if present, would bias our estimates in favor of larger impacts of the pandemic. We do not find evidence of recall bias. Mortality in months more proximate to the interview month is actually slightly lower than in months more distant (see tables S9 and S10), a pattern consistent with declining all-cause mortality in India before the pandemic (*18*). We also fail to find evidence that recall bias differs by sex or social group. Last, we show that mortality in the prepandemic months from January 2018 to May 2019 is similar across interview years of 2019, 2020, and 2021 in NFHS-5 (fig. S11). This suggests that mortality recall in NFHS-5 did not worsen as a result of the pandemic.

#### Validation of findings in CRS

In NFHS-5 states that have high rates of death registration for both females and males (see table S1), civil registration data show excess mortality patterns that are broadly consistent with the NFHS. As shown in fig. S3, excess mortality for females is higher than for males in all of the states that have high rates of death registration except for one, Chhattisgarh.

## Supplementary Materials

### This PDF file includes:

Tables S1 to S10 Figs. S1 to S11 References
