# Supplementary material for: Large and unequal life expectancy declines during the COVID-19 pandemic in India in 2020
Source: Sci Adv. 2024 Jul 19;10(29):eadk2070. doi: 10.1126/sciadv.adk2070 (PMC11259167; doi:10.1126/sciadv.adk2070)
Supplement: Supplementary file 1 — Tables S1 to S10 Figs. S1 to S11 References [file sciadv.adk2070_sm.pdf]

Science Advances
AAAS

Supplementary Materials for

Large and unequal life expectancy declines during the COVID-19 pandemic in India in 2020

Aashish Gupta *et al.*

Corresponding author: Sangita Vyas, sangita.vyas@hunter.cuny.edu;
Aashish Gupta, aashish.gupta@sociology.ox.ac.uk

Sci. Adv. **10**, eadk2070 (2024)
DOI: 10.1126/sciadv.adk2070

This PDF file includes:

Tables S1 to S10
Figs. S1 to S11
References

1 Description of SM Tables and Figures

Table S-1 shows rates of death registration for females and males separately in NFHS-5 subsample states. Death registration in union territories is not shown. Death registration rates are high for females and males in: Chhattisgarh, Madhya Pradesh, Punjab, Rajasthan, and Tamil Nadu. Death registration is low or unequal across females and males in: Arunachal Pradesh, Haryana, Jharkhand, Odisha, Uttar Pradesh and Uttarakhand.

Table S-2 calculates implied excess deaths for all of India, based on excess mortality observed during the pandemic months in the NFHS-5 subsample. We estimate 1.19 million excess deaths in 2020 nationally, and 0.33 million excess deaths in NFHS-5 subsample states and union territories. The table also documents a female disadvantage in excess deaths.

Table S-3 displays SARS-CoV-2 seroprevalence for India's states as measured by India's third national serosurvey, conducted between December 2020 and January 2021 (43). Seroprevalence at the state level is calculated as the mean prevalence in the districts of respective states. This serosurvey, which was conducted at the end of the period for which we report excess mortality estimates from the NHFS-5 subsample, suggests that seroprevalence in the subsample states was not different from the states not in the subsample.

Table S-4 shows observed deaths and person-years by year (2019 and 2020) in the NFHS-5 2021 subsample. Observed deaths and person-years account for survey weights. Person-years include estimated person-years lived by individuals who died in the respective year.

Tables S-5 through S-8 show that the NFHS-5 2021 subsample is similar in characteristics to the full NFHS-5 sample.

Tables S-9 and S-10 show results from linear regressions investigating if mortality is higher in calendar months closer to the month of interview. Months prior to interview are measured as negative numbers. For example, a value of -6 represents 6 months prior to interview. All regressions control for calendar month dummies (January, February, ...), which account for seasonal variation in mortality. Model (1) shows that months closer to interview month have slightly lower mortality compared to months further back, as would be expected with declines in overall mortality over time. Based on Model (1), we expect mortality to decline by 0.0022*12 deaths per 1,000 over a year, or by 0.0264 deaths per 1,000. This is relatively small compared to the crude death rate in a year. Model (2) shows that recall bias is not statistically different between males and females, and

Model (3) shows that it is not statistically different by social group.

Figure S-1 examines declines in life expectancy between 2019 and 2020. Estimates are shown by sex, social group, and the pooled subsample. Declines are highest among Muslims. They are also higher among females relative to males; and among other marginalized groups, such as Scheduled Castes and Scheduled Tribes, relative to high caste Hindus.

Figure S-2 shows that although overall declines in rural and urban areas are similar, declines in life expectancy between 2019 and 2020 are larger among rural males, rural females, and urban females compared to urban males.

Figure S-3 shows CRS 2020 excess mortality in NFHS-5 subsample states for which vital statistics are available. Estimates use 2019 mortality as baseline. Excess mortality is greater for females compared to males in all states that have high rates of death registration for females and males, except for Chhattisgarh. These results are broadly consistent with findings from the NFHS-5 subsample. CRS estimates of excess mortality in Arunachal Pradesh and Uttar Pradesh are not reliable because many deaths go unregistered in these states.

Figure S-4 shows results from an Arriaga decomposition, decomposing the age-specific contribution (for abridged life table age groups) of changes in life expectancy between 2019 and 2020 for males and females separately. For females, we see a large contribution of childhood ages (0-4), young adulthood (15-24), and older ages (50+). For males, we see a large contribution of childhood ages, and also of older working ages (40-59).

Figure S-5 compares excess mortality estimates from our subsample to excess mortality estimates from hypothetical sub-subsamples which exclude one subsample state each. We created 14 sub-subsamples. Excess mortality estimates observed in these hypothetical sub-subsamples is not statistically different from excess mortality in the subsample.

Figure S-6 shows annualized age-standardized crude death rates in the subsample by calendar month for the years 2018, 2019, and 2020. Pooled, rural, and urban mortality estimates are shown separately. Given that a larger share of the subsample consists of rural residents, confidence intervals around urban estimates are larger than those around rural estimates. In rural areas and in the overall subsample, we observe elevated mortality towards the end of the year. In urban areas, we observe elevated mortality in October 2020. Figure 6 shows that confirmed COVID-19 cases and deaths peaked in September 2020. A surge in cases around September 2020 is consistent with a

mortality peak slightly later in October 2020. Thus, one interpretation of Figure S-6 is that case and death data tracked the epidemic in urban areas better. Data on confirmed COVID-19 cases and deaths fails to capture elevated mortality in rural areas towards the end of the year.

Figure S-7 shows the distribution of NFHS-5 interview by survey phase. Most Phase 1 interviews were conducted between July 2019 and December 2019. Phase 2 interviews began before the first national lockdown and were conducted largely between January and March 2020. Interviews resumed in October 2020, but the vast majority of interviews were conducted between December 2020 and April 2021. Interviews in January 2021 and after, shown as blue bars, comprise our analytical subsample.

Figure S-8 shows the distribution of NFHS-5 interviews by state. Related to this, Figure S-9 shows the spatial distribution of Primary Sampling Units (PSU) in our subsample in blue. PSUs that were interviewed before 2021 are shown in red.

Figure S-10 shows age-specific mortality rates for 2019 and 2020 separately using the NFHS-5 2021 subsample. Mortality rates in 2020 are higher than mortality rates in 2019 at all ages. Although confidence intervals sometimes overlap, Figure 3 shows that 95% confidence intervals for differences between 2019 and 2020 age-specific rates do not contain zero at the youngest and older ages.

Figure S-11 shows crude death rates for January 2018 to May 2019 by year of interview. Seasonal influences on mortality, phased implementation of NFHS-5 whereby states and districts were interviewed in different periods, and recall errors can result in variation in mortality estimates by calendar-month and survey-year. There are no systematic differences in estimated crude death rates by interview year. Recall bias does not appear to influence estimates from 2021 interviews. Additionally, the NFHS-5 2021 subsample does not show systematically different crude death rates compared to the rest of the NFHS-5 subsample.

Table S-1: Death registration in NFHS-5 2021 subsample states

NFHS-5 subsample state	Proportion of deaths registered		CRS 2019 & 2020 available
	Male	Female	
	(1)	(2)	(3)
Arunachal Pradesh	0.55	0.48	yes
Chhattisgarh	0.84	0.83	yes
Haryana	0.90	0.83	no
Jharkhand	0.47	0.37	no
Madhya Pradesh	0.86	0.81	yes
Odisha	0.77	0.65	yes
Punjab	0.96	0.94	yes
Rajasthan	0.82	0.80	yes
Tamil Nadu	0.95	0.93	yes
Uttar Pradesh	0.59	0.47	yes
Uttarakhand	0.87	0.68	no

Notes: Death registration statistics are shown for NFHS-5 2021 subsample states. Deaths between January 2017 and May 2019, when the survey started collecting data, are included. Column (3) notes whether data for the state is available from the Civil Registration System in both 2019 and 2020. Source: NFHS-5.

Table S-2: National and subsample estimates of excess mortality in India

	Female (1)	Male (2)	Pooled (3)
1. Observed excess mortality P-scores, (%)	21.4	14.2	17.1
(Apr - Dec 2020, compared to 2019 baseline)	[10.8, 32.0]	[5.5, 22.9]	[10.5, 23.7]
2. Annual number of deaths, 2019 (millions)	4.28	5.00	9.28
(UN WPP Estimate)			
3. Implied excess deaths nationally (millions)	0.69	0.53	1.19
(Apr - Dec 2020, compared to 2019 baseline)	[0.35, 1.03]	[0.21, 0.86]	[0.73, 1.65]
4. Increase in Crude Death Rate (per 1,000)	1.4	1.29	1.35
($\text{AASCDR}^{Apr-Dec2020}$ – $\text{AASCDR}^{Apr-Dec2019}$)	[0.78, 2.07]	[0.55, 2.03]	[0.87, 1.83]
5. Implied excess deaths in subsample (millions)	0.17	0.16	0.33
(Apr - Dec 2020, compared to 2019 baseline)	[0.09, 0.24]	[0.07, 0.25]	[0.21, 0.44]

Note: Row 1 of the table shows observed percentage increase in mortality in April through December 2020 (the 2020 pandemic months), relative to the same months in 2019, in the NFHS-5 2021 subsample. Row 2 shows annual estimates of deaths in India nationally in 2019 from the UN World Population Prospects (18). Row 3 shows the implied excess deaths nationally if excess mortality from the NFHS-5 2021 subsample were observed nationally. Row 3 is calculated based on the following formula: $\frac{row1 \times row2 \times 0.75}{100}$. Row 4 shows the observed increase in Annualized Age-Standardized Cride Death Rates (AASCDR) in the pandemic months of 2020 compared to the same months in 2019. Row 5 estimates the implied excess deaths based on the observed increase in the crude death rate for the NFHS-5 subsample. The calculation assumes that the subsample is representative of 23.15% of India, and uses female, male, and total population estimates from the UN WPP (18). 95% confidence intervals for NFHS-5 estimates are shown in brackets below estimates and are calculated using a cluster-bootstrap approach. Sources: NFHS-5, UN WPP 2022.

Table S-3: Seroprevalence from India's third national serosurvey at the end of 2020 is similar in NFHS-5 2021 subsample states and states interviewed by NFHS before 2021

	Seroprevalence (%) (Dec 2020 - Jan 2021)	Population (000) (2020 estimate)
States in NFHS-5 2021 Subsample		
Chhattisgarh	24.2	29,109
Haryana	21.0	29,077
Jharkhand	21.3	37,937
Madhya Pradesh	22.8	83,374
Odisha	33.9	45,350
Punjab	19.8	30,099
Rajasthan	27.5	78,273
Tamil Nadu	28.8	76,049
Uttar Pradesh	23.3	227,943
Uttarakhand	12.0	11,270
Estimated seroprevalence in subsample	24.6	
States interviewed by NFHS-5 before 2021		
Andhra Pradesh	35.1	52,504
Assam	32.1	34,668
Bihar	29.5	121,302
Gujarat	22.1	68,862
Himachal Pradesh	46.5	7,347
Jammu and Kashmir	32.0	13,305
Karnataka	29.3	66,322
Kerala	9.7	35,307
Maharashtra	21.6	123,295
Telangana	29.5	37,473
West Bengal	28.1	97,516
Estimated seroprevalence out of subsample	26.8	

Note: Seroprevalence is based on estimates of India's third national serosurvey, conducted in December 2020 and January 2021 (43). Estimates for Union Territories are not available from the serosurvey. Overall seroprevalence was estimated to be 24.1% [95% CI: 23.0-25.3]. Population estimates for 2020 are from population projections by India's National Commission of Population (84). Overall seroprevalence in NFHS-5 2021 subsample and out-of-subsample states is estimated as a weighted average of state-level seroprevalence and population estimates. Serosurveyed districts in each state were not chosen to be representative of the population of that state. Sources: (43), (84).

Table S-4: Observed deaths and person-years in NFHS-5 subsample

Group	2019		2020	
	Deaths	Person-Years	Deaths	Person-Years
		Overall		
Overall	4,700	620,211	5,773	626,044
		By Sex		
Female	1,949	310,882	2,546	313,950
Male	2,751	309,329	3,228	312,094
		By Social Group		
Scheduled Castes	1,168	143,122	1,442	144,524
Scheduled Tribes	415	64,740	553	65,587
Muslims	391	66,224	586	67,230
Other Backward Classes	1,861	237,825	2,143	239,766
High castes	701	87,387	820	87,940
Others	165	20,912	229	20,998
		By Abridged Life Table Age Groups		
0	359	9,164	421	9,195
1	51	42,317	56	41,360
5	30	58,143	37	58,137
10	35	61,118	38	60,270
15	67	59,716	117	60,734
20	95	56,149	135	55,747
25	102	50,987	115	53,134
30	100	45,326	109	44,462
35	118	40,173	135	42,167
40	126	36,947	178	36,293
45	207	31,553	213	33,558
50	227	34,862	325	33,374
55	305	30,307	389	30,747
60	409	25,274	487	26,161
65	445	15,807	548	17,046
70	570	10,086	690	10,788
75	368	5,823	471	6,173
80	426	3,405	453	3,586
85+	662	3,054	857	3,111

Notes: The table shows observed deaths and person-years by year (2019 and 2020) in the NFHS-5 2021 subsample. Observed deaths and person-years account for survey weights. Person-years include estimated person-years lived by individuals who died in the respective year. Source: NFHS-5.

Table S-5: Comparison of NFHS-5 2021 subsample (the main analysis sample) with the full NFHS-5 sample on household socio-economic characteristics

	Subsample (Only 2021 interviews)	Full sample	Difference
	(1)	(2)	(1) - (2)
Rural	0.67	0.67	-0.00
	(0.01)	(0.00)	(0.01)
Religion			
Hindu	0.84	0.81	0.03
	(0.00)	(0.00)	(0.00)
Muslim	0.10	0.13	-0.03
	(0.00)	(0.00)	(0.00)
Christian	0.02	0.03	-0.01
	(0.00)	(0.00)	(0.00)
Sikh	0.03	0.01	0.01
	(0.00)	(0.00)	(0.00)
Other	0.01	0.01	-0.01
	(0.00)	(0.00)	(0.00)
Caste			
Scheduled Caste	0.23	0.23	0.00
	(0.00)	(0.00)	(0.00)
Scheduled Tribe	0.11	0.10	0.01
	(0.00)	(0.00)	(0.00)
Other Backward Class	0.48	0.43	0.06
	(0.00)	(0.00)	(0.01)
Other	0.18	0.25	-0.07
	(0.00)	(0.00)	(0.00)
Wealth Index			
Poorest	0.22	0.20	0.02
	(0.00)	(0.00)	(0.00)
Poorer	0.19	0.20	-0.02
	(0.00)	(0.00)	(0.00)
Middle	0.18	0.21	-0.03
	(0.00)	(0.00)	(0.00)
Richer	0.18	0.20	-0.02
	(0.00)	(0.00)	(0.00)
Richest	0.23	0.19	0.04
	(0.00)	(0.00)	(0.01)

Notes: Column (1) of the table gives summary statistics of the NFHS-5 2021 subsample. Column (2) displays summary statistics using the full NFHS-5 sample. Column (3) reports the difference between the two. Observations are households. The wealth index is computed by The DHS Program, and is based on household ownership of assets such as motorcycle or television; dwelling characteristics such as flooring material; type of drinking water source; toilet facilities; and type of cooking fuel. Estimates use sample weights. Clustered standard errors are reported in parentheses under each estimate. Source: NFHS-5.

Table S-6: Comparison of NFHS-5 2021 subsample (the main analysis sample) with the full NFHS-5 sample on individual demographic characteristics

	Subsample (Only 2021 interviews)	Full sample	Difference
	(1)	(2)	(1) - (2)
Males by age			
0-5	0.16	0.14	0.02
	(0.00)	(0.00)	(0.00)
6-20	0.27	0.26	0.00
	(0.00)	(0.00)	(0.00)
21-40	0.28	0.29	-0.01
	(0.00)	(0.00)	(0.00)
41-60	0.19	0.20	-0.01
	(0.00)	(0.00)	(0.00)
61-80	0.10	0.10	-0.00
	(0.00)	(0.00)	(0.00)
80+	0.01	0.01	0.00
	(0.00)	(0.00)	(0.00)
Females by age			
0-5	0.14	0.13	0.02
	(0.00)	(0.00)	(0.00)
6-20	0.26	0.25	0.01
	(0.00)	(0.00)	(0.00)
21-40	0.29	0.30	-0.01
	(0.00)	(0.00)	(0.00)
41-60	0.21	0.21	-0.01
	(0.00)	(0.00)	(0.00)
61-80	0.09	0.09	-0.00
	(0.00)	(0.00)	(0.00)
80+	0.01	0.01	0.00
	(0.00)	(0.00)	(0.00)
Population Coverage(%)	23.15	100	–
N	765,180	3,002,140	–

Notes: Column (1) of the table gives summary statistics of the NFHS-5 2021 subsample. Column (2) displays summary statistics using the full NFHS-5 sample. Column (3) reports the difference between the two. Observations are individuals, including those who were reported to have died, and those who were alive at the time of interview. Age groups are constructed using the last observed age of individuals. For deceased individuals, this is the age at death, whereas for those who were alive, it is the age at the time of the interview. Estimates use sample weights. Clustered standard errors are reported in parentheses under each estimate. Source: NFHS-5.

Table S-7: Comparison of NFHS-5 2021 subsample (the main analysis sample) with the full NFHS-5 sample for individuals alive at the time of the survey

	Subsample (Only 2021 interviews)	Full sample	Difference
	(1)	(2)	(1) - (2)
HH Size	5.73	5.42	0.31
	(0.02)	(0.01)	(0.02)
Years of Education	6.04	5.94	0.10
	(0.03)	(0.02)	(0.04)
Household owns			
Electricity	0.96	0.97	-0.00
	(0.00)	(0.00)	(0.00)
Radio	0.03	0.05	-0.02
	(0.00)	(0.00)	(0.00)
Television	0.67	0.69	-0.02
	(0.00)	(0.00)	(0.00)
Refrigerator	0.41	0.38	0.03
	(0.00)	(0.00)	(0.01)
Bicycle	0.62	0.53	0.09
	(0.00)	(0.00)	(0.00)
Motorcycle/scooter	0.61	0.52	0.09
	(0.00)	(0.00)	(0.00)
Car/truck	0.08	0.08	0.00
	(0.00)	(0.00)	(0.00)
Mobile phone	0.95	0.96	-0.00
	(0.00)	(0.00)	(0.00)
Livestock	0.51	0.46	0.05
	(0.01)	(0.00)	(0.01)

Notes: Column (1) of the table gives summary statistics of the NFHS-5 2021 subsample. Column (2) displays summary statistics using the full NFHS-5 sample. Column (3) reports the difference between the two. Observations are individuals who were alive at the time of the survey. Estimates use sample weights. Clustered standard errors are reported in parentheses under each estimate. Source: NFHS-5.

Table S-8: Comparison of NFHS-5 2021 subsample (the main analysis sample) with the full NFHS-5 sample on mortality-related characteristics

	Subsample (Only 2021 interviews)	Full sample	Difference
	(1)	(2)	(1) - (2)
Has Toilet	0.81	0.81	-0.00
	(0.00)	(0.00)	(0.00)
Access to Piped Water	0.29	0.35	-0.06
	(0.00)	(0.00)	(0.01)
Cooks with Clean Fuel	0.50	0.58	-0.07
	(0.01)	(0.00)	(0.01)
Individuals > 15 Years of Age			
Drinks Alcohol	0.10	0.10	0.00
	(0.00)	(0.00)	(0.00)
Smokes Tobacco	0.22	0.23	-0.01
	(0.00)	(0.00)	(0.00)
BP (Systolic)	125.58	123.86	1.71
	(0.08)	(0.05)	(0.09)
BP (Diastolic)	87.62	86.52	1.10
	(0.05)	(0.03)	(0.06)
Children < 5 Years of Age			
Height for Age (SD)	-1.21	-1.24	0.03
	(0.01)	(0.01)	(0.01)
Weight for Age (SD)	-1.44	-1.53	0.09
	(0.01)	(0.01)	(0.01)
On-track with immunizations at age 1	0.73	0.73	0.00
	(0.01)	(0.00)	(0.01)
Hospital/Clinic/Facility Birth	0.89	0.88	0.01
	(0.00)	(0.00)	(0.00)

Notes: Proportion of children under age 5 who were on track with immunizations at age 1 is computed based on the Indian government's immunization schedule for children. Column (1) of the table gives summary statistics of the NFHS-5 2021 subsample. Column (2) displays summary statistics using the full NFHS-5 sample. Column (3) reports the difference between the two. Observations are individuals who were alive at the time of the survey. Estimates use sample weights. Clustered standard errors are reported in parentheses under each estimate. Source: NFHS-5.

Table S-9: Robustness check: No evidence of recall bias in NFHS-4 (2015-16)

dependent variable:	monthly crude death rate, per 1,000		
	(1)	(2)	(3)
months prior to interview	-0.0022***	-0.0022***	-0.0016
	(0.0003)	(0.0005)	(0.0009)
female X months prior to interview		0.0001	
		(0.0007)	
female		-0.1522***	
		(0.0115)	
Scheduled Caste X months prior to interview			-0.0007
			(0.0012)
Scheduled Tribe X months prior to interview			-0.0015
			(0.0011)
Other Backward Class X months prior to interview			0.0008
			(0.0014)
Muslim X months prior to interview			-0.0003
			(0.0010)
Others X months prior to interview			-0.0022
			(0.0026)
Scheduled Caste			0.0046
			(0.0206)
Scheduled Tribe			-0.0899***
			(0.0201)
Other Backward Class			-0.1739***
			(0.0253)
Muslim			-0.0248
			(0.0178)
Others			0.0179
			(0.0457)
Dummies for calendar month	X	X	X
Constant	0.6542***	0.7296***	0.6890***
	(0.0106)	(0.0125)	(0.0175)
N	76,912,746	76,912,746	76,912,746

Notes: The table shows results from linear regressions investigating if mortality is higher in calendar months closer to the month of interview. A death is coded as 1,000, implying that the dependent variable corresponds to the monthly crude death rate, per 1,000. Months prior to interview are measured as negative numbers. For example, a value of -6 represents 6 months prior to interview. We restrict the data to 30 months prior to the interview month. Model (2) includes interactions of female with months prior to interview, and model (3) include interactions of social group dummies with months prior to interview. All regressions control for calendar month dummies (January, February, ...), which account for seasonal variation in mortality. Standard errors, clustered at the level of primary sampling unit, are reported in parentheses under each estimate. Estimates use sample weights. * $p < 0.05$, ** $p < 0.01$, *** $p < 0.001$. Source: NFHS-4.

Table S-10: Robustness check: No evidence of recall bias in NFHS-5 pre-pandemic interviews (June 2019 - March 2020)

dependent variable:	monthly crude death rate, per 1,000		
	(1)	(2)	(3)
months prior to interview	-0.0004	-0.0001	0.0006
	(0.0004)	(0.0006)	(0.0010)
female X months prior to interview		-0.0006	
		(0.0008)	
female		-0.1661***	
		(0.0137)	
Scheduled Caste X months prior to interview			-0.0033*
			(0.0014)
Scheduled Tribe X months prior to interview			0.0005
			(0.0014)
Other Backward Class X months prior to interview			0.0022
			(0.0015)
Muslim X months prior to interview			-0.0017
			(0.0013)
Others X months prior to interview			-0.0028
			(0.0017)
Scheduled Caste			0.0569*
			(0.0250)
Scheduled Tribe			-0.1420***
			(0.0242)
Other Backward Class			-0.2170***
			(0.0269)
Muslim			0.0278
			(0.0227)
Others			-0.0667*
			(0.0307)
Dummies for calendar month	X	X	X
Constant	0.6725***	0.7560***	0.7048***
	(0.0138)	(0.0159)	(0.0221)
N	56,416,345	56,416,345	56,416,345

Notes: The table shows results from linear regressions investigating if mortality is higher in calendar months closer to the month of interview. A death is coded as 1,000, implying that the dependent variable corresponds to the monthly crude death rate, per 1,000. Months prior to interview are measured as negative numbers. For example, a value of -6 represents 6 months prior to interview. We restrict the data to 30 months prior to the interview month. Model (2) includes interactions of female with months prior to interview, and model (3) include interactions of social group dummies with months prior to interview. All regressions control for calendar month dummies (January, February, ...), which account for seasonal variation in mortality. Standard errors, clustered at the level of primary sampling unit, are reported in parentheses under each estimate. Estimates use sample weights. * $p < 0.05$, ** $p < 0.01$, *** $p < 0.001$. Source: NFHS-5.

Figure S-1: Decline in period life expectancy at birth between 2019 and 2020 by sex and social group

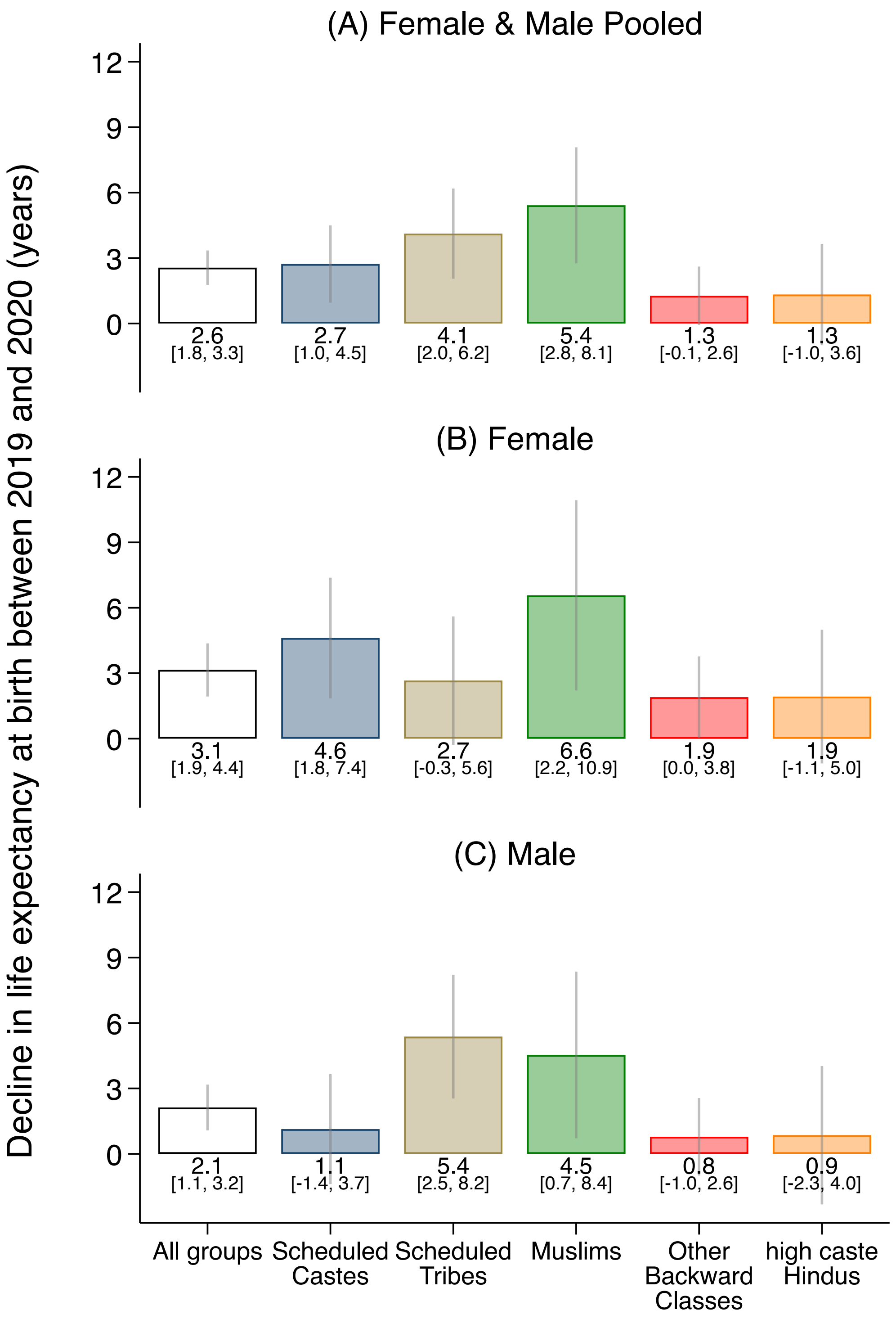

Note: The figure shows declines in life expectancy at birth by social group between 2019 and 2020 for (A) the combined female and male population, (B) females, and (C) males, separately. Life expectancy is calculated based on standard life table procedures. Estimates are for the NFHS-5 2021 subsample and use sample weights. The vertical lines around each estimate represent 95% confidence intervals calculated using a cluster-bootstrap approach. Source: NFHS-5.

Figure S-2: Period life expectancy at birth in 2019 and 2020 by sex and residence

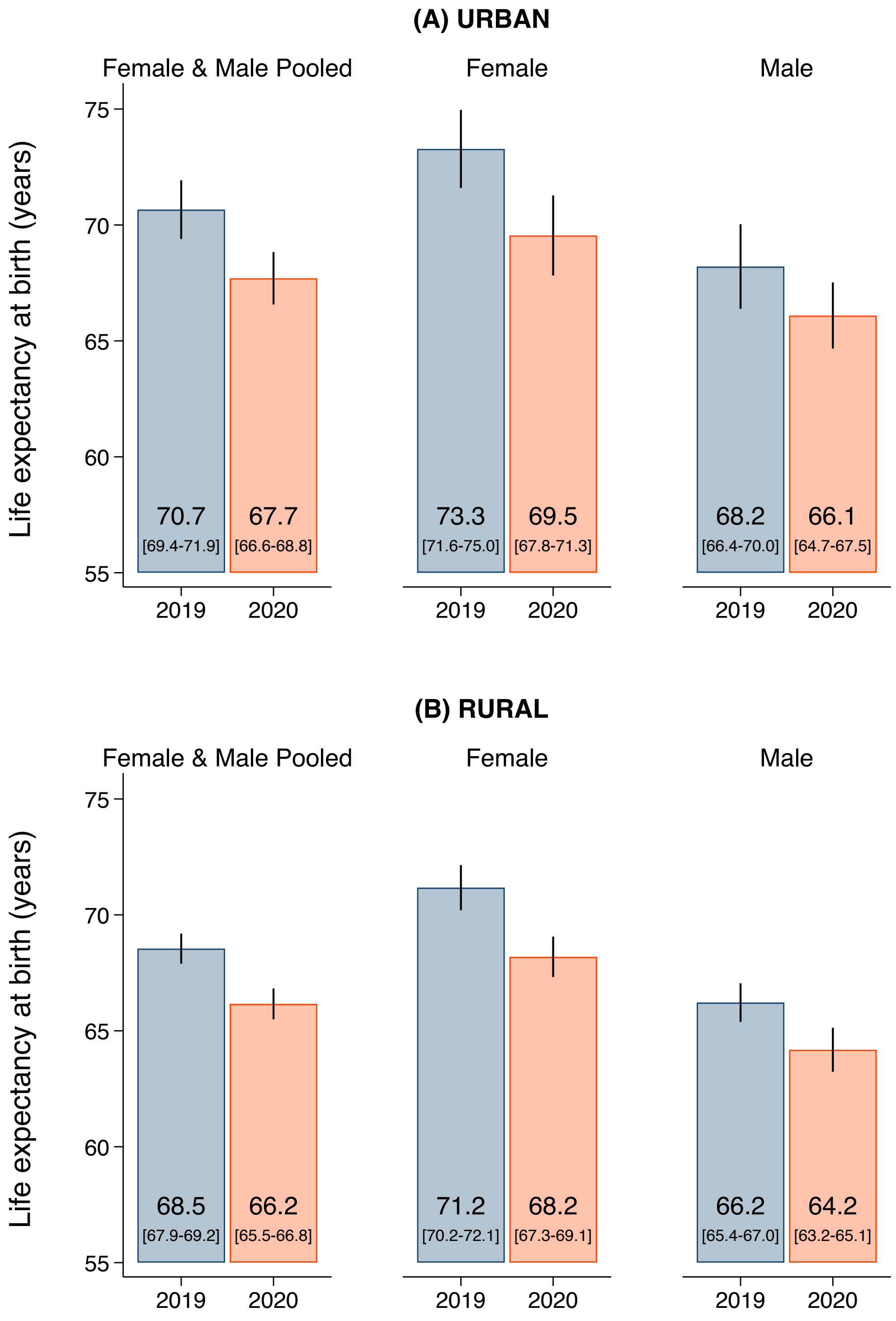

Note: The figure shows life expectancy at birth in 2019 and 2020 for (A) the urban population and (B) the rural population. In each panel, estimates are shown for the combined female and male population, for females, and males, separately. Life expectancy is calculated based on standard life table procedures. Estimates are for the NFHS-5 2021 subsample and use sample weights. The vertical lines around each estimate represent 95% confidence intervals calculated using a cluster-bootstrap approach. Source: NFHS-5.

Figure S-3: Robustness check: 2020 excess mortality by sex and state in the Civil Registration System, for NFHS-5 2021 subsample states

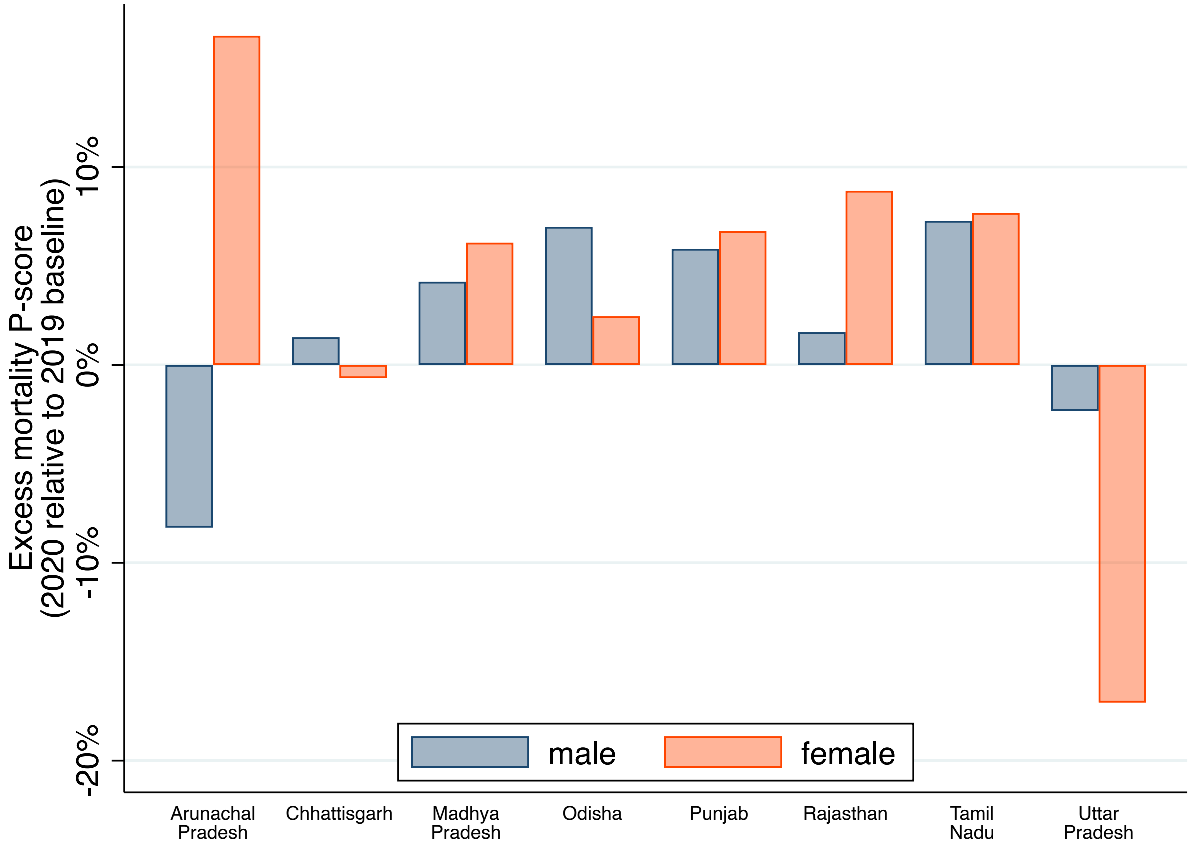

Note: CRS excess mortality is shown for NFHS-5 subsample states for which CRS data are available. Excess mortality is computed as the percent increase in mortality between 2019 and 2020. Greater increases in mortality are observed among females compared to males in all states that have high rates of death registration for females and males, except for Chhattisgarh. See Table S-1 for rates of death registration by sex and state. Source: Civil Registration System.

Figure S-4: Age-specific contributions (by abridged life table age groups) to changes in period life expectancy at birth between 2019 and 2020

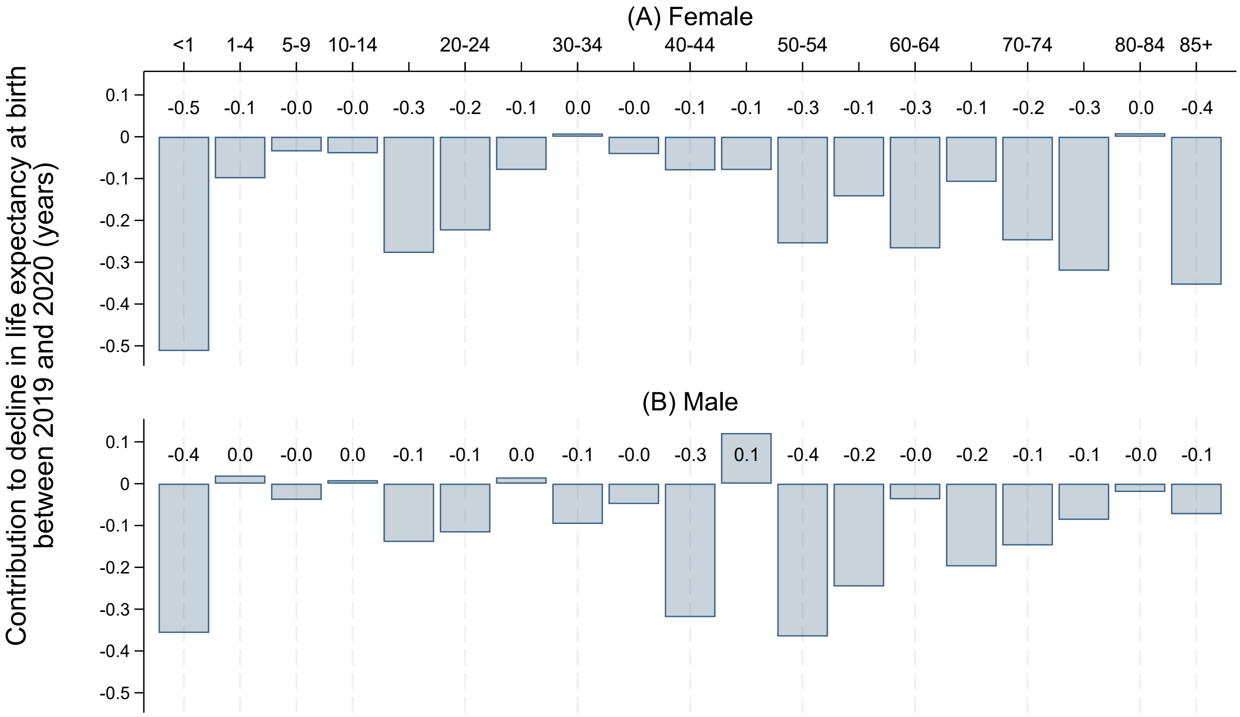

Note: The figure shows the decomposition of the change in life expectancy between 2019 and 2020 into contributions from changes in mortality in different age groups based on Arriaga's decomposition for (A) females and (B) males (79). Estimates are for the NFHS-5 2021 subsample and use sample weights. Source: NFHS-5.

Figure S-5: Robustness check: Excess mortality estimate is robust to excluding one state at a time in hypothetical sub-subsamples

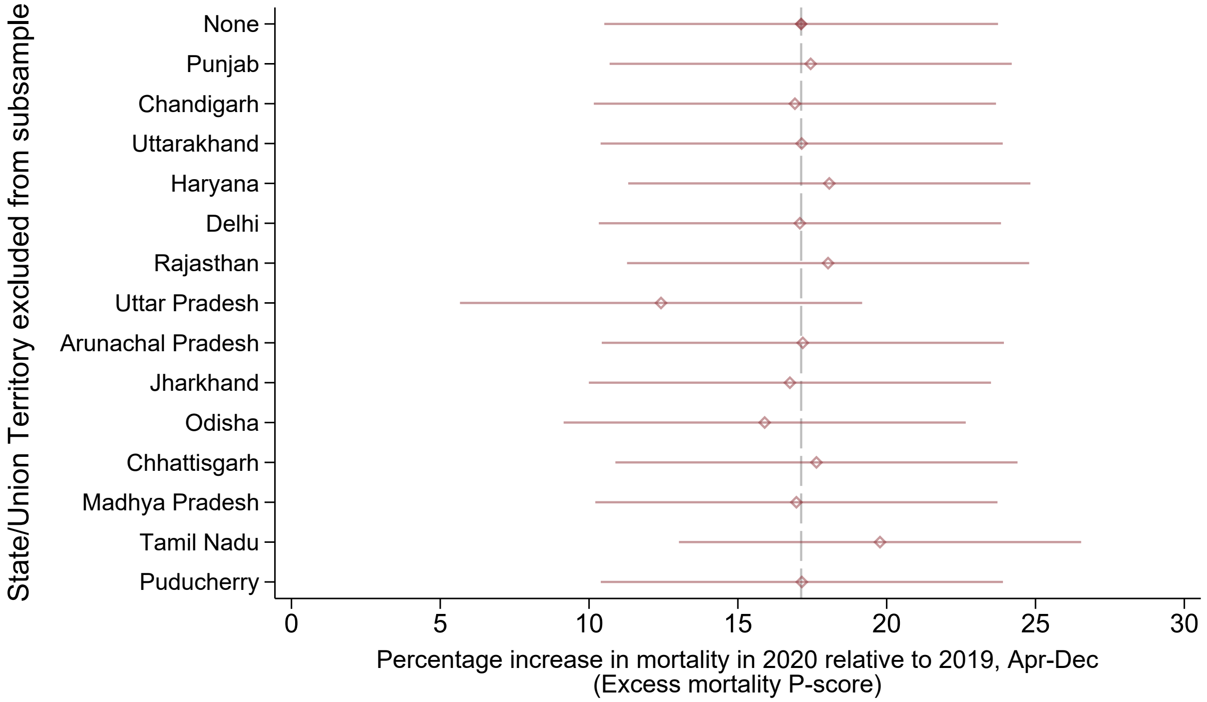

Note: The figure shows results from an analysis which estimates the excess mortality P-score from hypothetical sub-subsamples (subsamples of the NFHS-5 2021 subsample) that exclude one state at a time from the subsample. Excess mortality P-scores are the percentage increase in mortality in April through December 2020 relative to the same months in 2019. Estimates use sample weights, and 95% confidence intervals calculated using a cluster-bootstrap approach are shown as horizontal lines around estimates. Source: NFHS-5.

Figure S-6: Crude death rates by calendar month and place of residence, NFHS-5 2021 interviews subsample

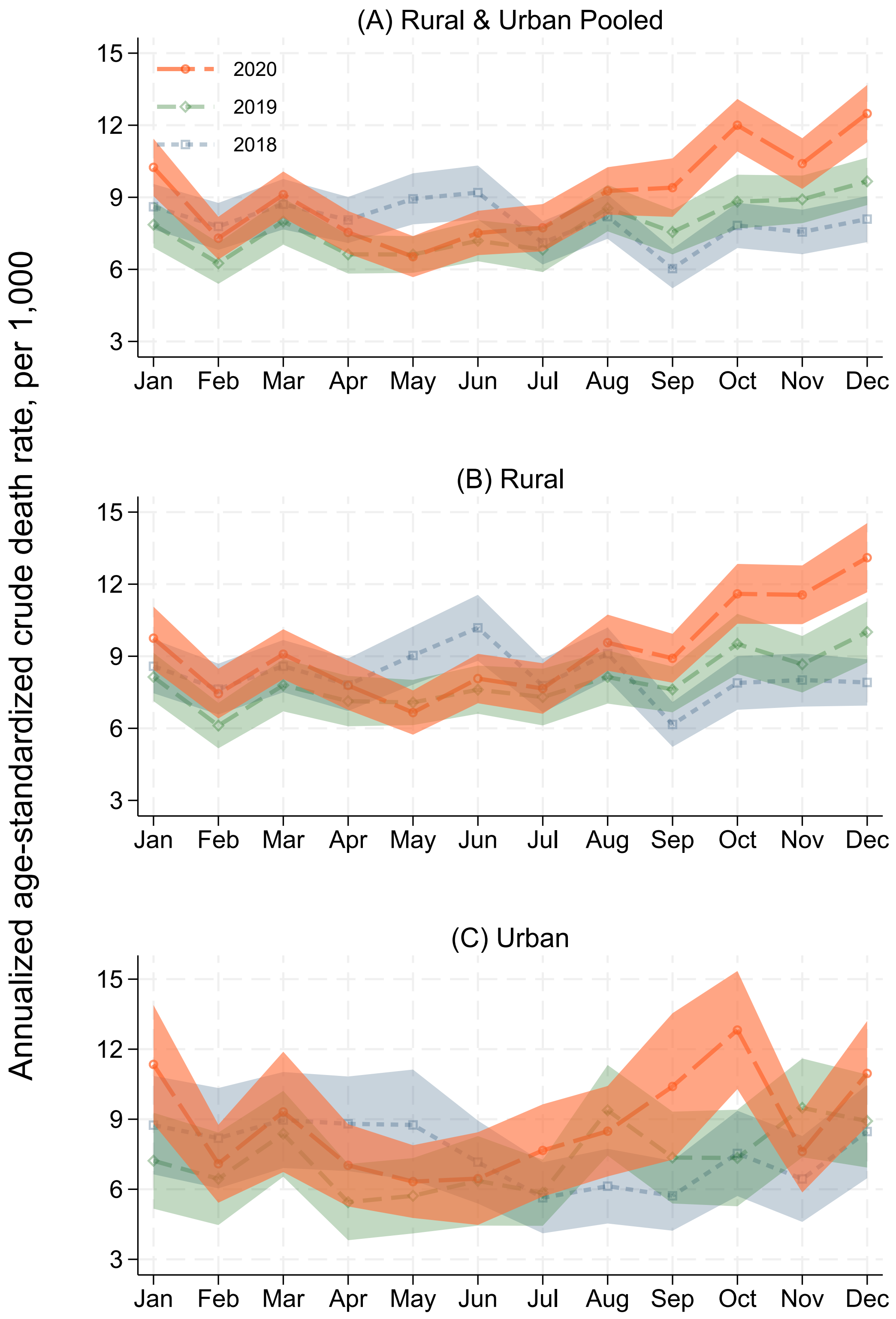

Note: Monthly crude death rates for (A) the combined rural and urban population, (B) the rural population, and (C) the urban population, separately. Estimates are for the NFHS-5 2021 subsample and use sample weights. Monthly crude death rates are age-standardized and annualized. 95% confidence intervals are shown as the shaded area around estimates and are calculated using a cluster-bootstrap approach. Source: NFHS-5.

Figure S-7: Proportion of NFHS-5 interviews conducted in each calendar month in each survey phase

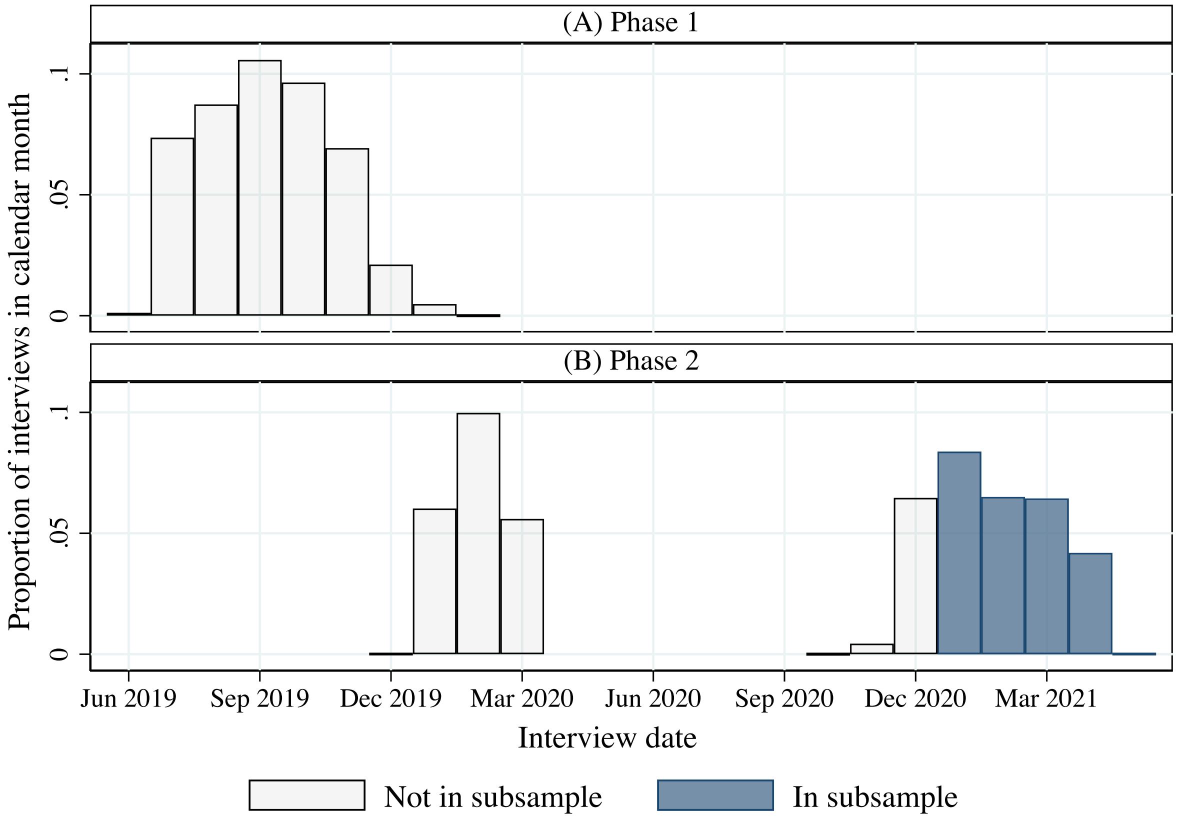

Note: The figure shows the proportion of NFHS-5 interviews carried out in each calendar month in (A) Phase 1 and (B) Phase 2. The main analysis sample used in this paper includes households interviewed in 2021, shown in blue. Source: NFHS-5.

Figure S-8: Proportion of NFHS-5 interviews conducted in each calendar month in each state or union territory between 2019 and 2021

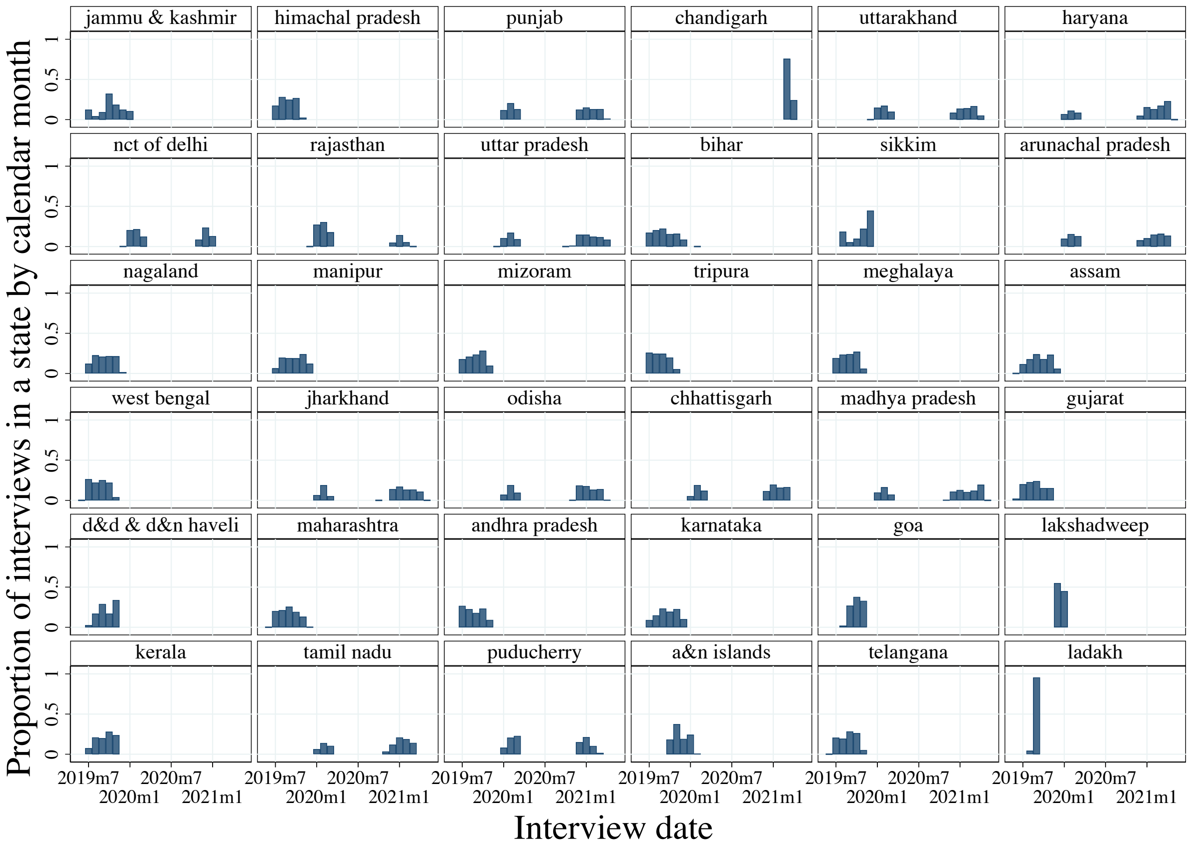

Note: The figure shows the proportion of NFHS-5 interviews carried out in each calendar month in each state. The main analysis sample used in this paper includes households interviewed in 2021. Source: NFHS-5.

Figure S-9: Spatial distribution of Primary Sampling Units (Clusters) in NFHS-5 2021 subsample

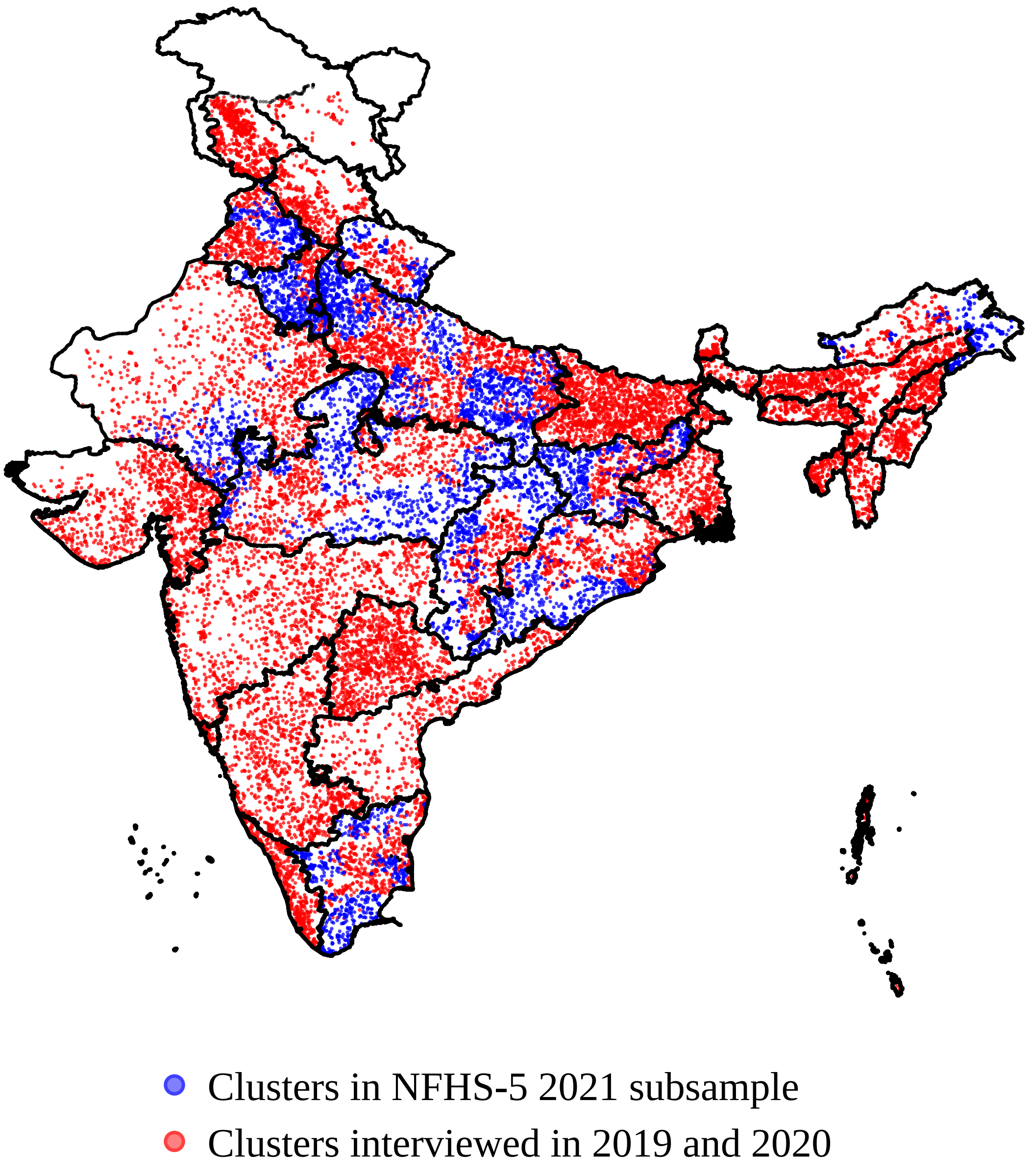

Note: Primary Sampling Units in the NFHS-5 2021 subsample are shown in blue, and Primary Sampling Units interviewed in 2019 and 2020 (and therefore not in the NFHS-5 2021 subsample), are shown in red. Source: NFHS-5.

Figure S-10: Age-specific mortality rates, 2019 and 2020, NFHS-5 2021 subsample

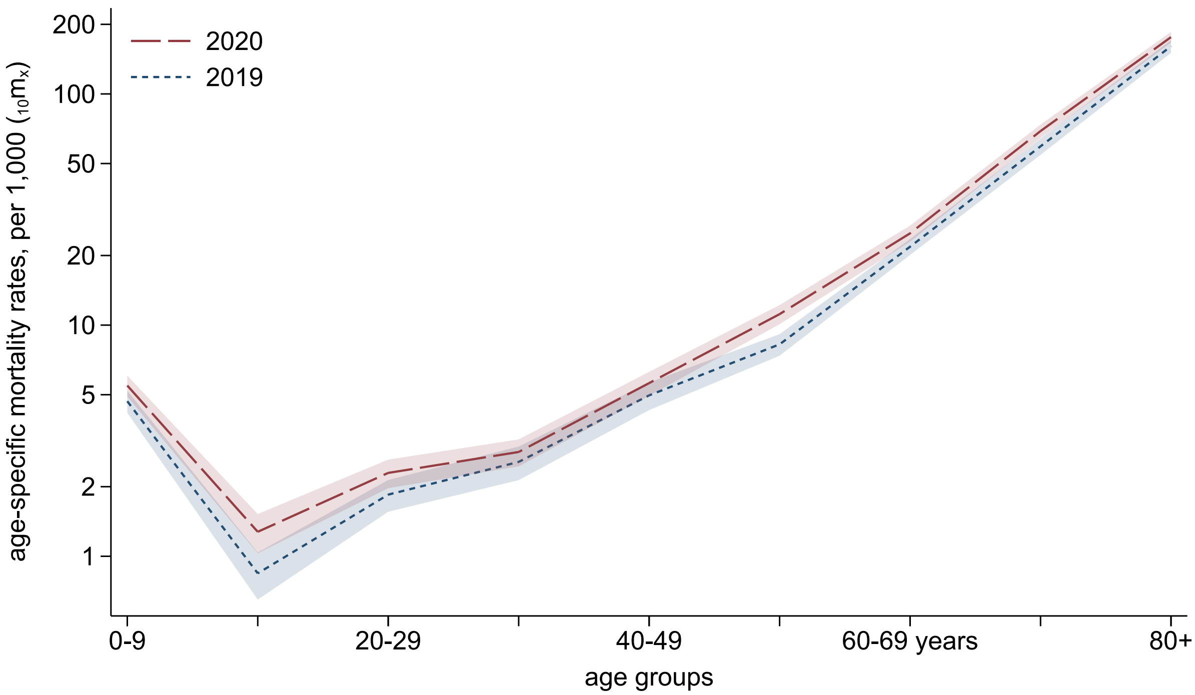

Note: The figure shows age-specific mortality rates, in ten-year age intervals, for 2019 and 2020, as observed in the NFHS-5 2021 subsample. Estimates use sample weights. 95% confidence intervals calculated using a cluster-bootstrap approach are shown as the shaded area around each line. Source: NFHS-5.

Figure S-11: Robustness check: Death rates for the months January 2018 to May 2019 by year of interview in NFHS-5 (2019-21)

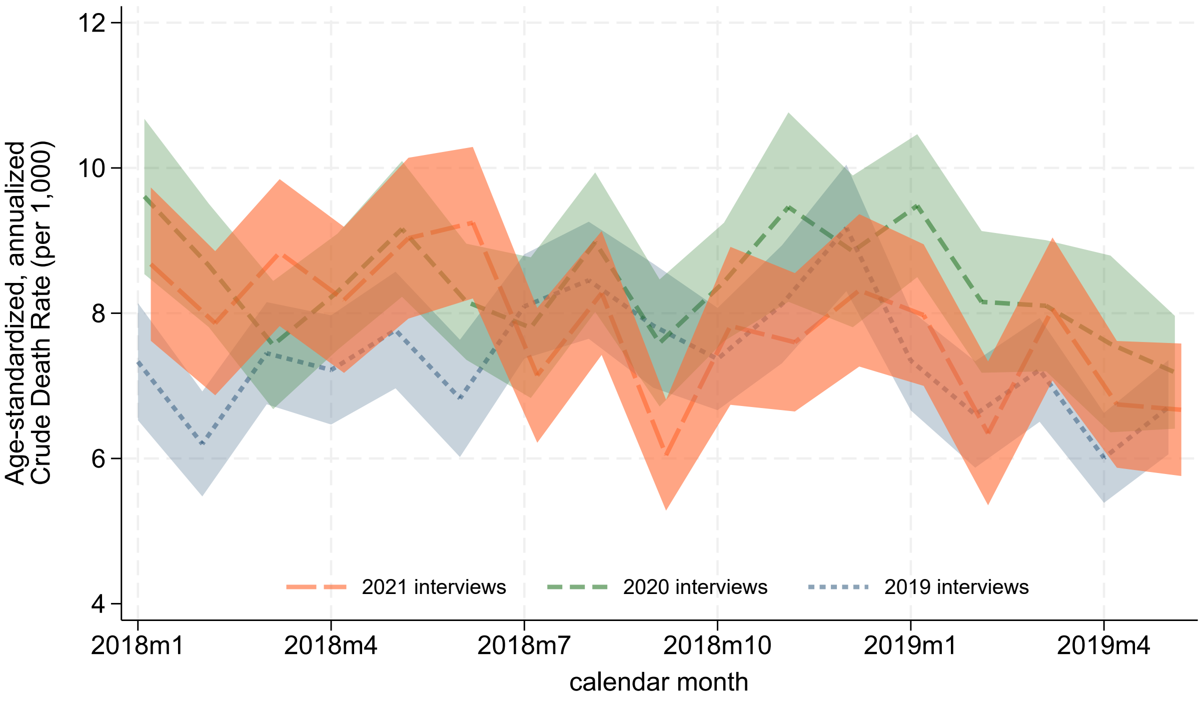

Note: The figure displays monthly crude death rates, for calendar months January 2018 to May 2019, by year of interview. Data are from the full NFHS-5 sample. Estimates use sample weights. Monthly crude death rates are age-standardized and annualized. The vertical lines around each estimate represent 95% confidence intervals calculated using a cluster-bootstrap approach. Source: NFHS-5.

REFERENCES AND NOTES

1. J. Schöley, J. M. Aburto, I. Kashnitsky, M. S. Kniffka, L. Zhang, H. Jaadla, J. B. Dowd, R. Kashyap, Life expectancy changes since COVID-19. *Nat. Hum. Behav.* **6**, 1649–1659 (2022).

2. J. M. Aburto, A. M. Tilstra, G. Floridi, J. B. Dowd, Significant impacts of the COVID-19 pandemic on race/ethnic differences in US mortality. *Proc. Natl. Acad. Sci. U.S.A.* **119**, e2205813119 (2022).

3. T. Andrasfay, N. Goldman, Reductions in 2020 US life expectancy due to COVID-19 and the disproportionate impact on the Black and Latino populations. *Proc. Natl. Acad. Sci. U.S.A.* **118**, e2014746118 (2021).

4. L. Cuéllar, I. Torres, E. Romero-Severson, R. Mahesh, N. Ortega, S. Pungitore, R. Ke, N. Hengartner, Excess deaths reveal unequal impact of COVID-19 in Ecuador. *BMJ Glob. Health* **6**, e006446 (2021).

5. A. Case, A. Deaton, "Accounting for the widening mortality gap between adult Americans with and without a BA" (Tech. rep. National Bureau of Economic Research Cambridge, Mass., USA, 2023).

6. A. N. Luck, S. H. Preston, I. T. Elo, A. C. Stokes, The unequal burden of the Covid-19 pandemic: Capturing racial/ethnic disparities in US cause-specific mortality. *SSM-Popul. Health* **17**, 101012 (2022).

7. S. Helleringer, B. L. Queiroz, Commentary: Measuring excess mortality due to the COVID-19 pandemic: Progress and persistent challenges. *Int. J. Epidemiol.* **51**, 85–87 (2022).

8. P. Heuveline, Global and national declines in life expectancy: An end-of-2021 assessment. *Popul. Dev. Rev.* **48**, 31–50 (2022).

9. J. Y. Ho, "What demographers need–and what the world needs from demographers–in response to covid-19" (Tech. rep., Population Council, 2021).

10. M. R. Nepomuceno, E. Acosta, D. Alburez-Gutierrez, J. M. Aburto, A. Gagnon, C. M. Turra , Besides population age structure, health and other demographic factors can contribute to understanding the COVID-19 burden. *Proc. Natl. Acad. Sci. U.S.A.* **117**, 13881–13883 (2020).

11. W. Msemburi, A. Karlinsky, V. Knutson, S. Aleshin-Guendel, S. Chatterji, J. Wakefield , The WHO estimates of excess mortality associated with the COVID-19 pandemic. *Nature* **613**, 130–137 (2023).

12. M. Banaji, A. Gupta, V. Paikra, Mortality and death registration in India: The many holes in the data. *The India Forum* (2022).

13. C. Z. Guilmoto, An alternative estimation of the death toll of the Covid-19 pandemic in India. *PLOS ONE* **17**, e0263187 (2022).

14. J. P. Drèze, A. Somanchi, "Not having the have-nots," *Economic Times*, 21 June 2021.

15. S. Rukmini, "India's COVID-19 count could be missing many women," *LiveMint*, 29 August 2020.

16. A. Gupta, "Health, environment, and inequality in India," thesis, University of Pennsylvania, Philadelphia, PA (2021).

17. A. Gupta, N. Sudharsanan, Large and persistent life expectancy disparities between India's social groups. *Popul. Dev. Rev.* **48**, 863–882 (2022).

18. United Nations Department of Economic and Social Affairs, Population Division, *World Population Prospects 2022: Summary of Results* (United Nations Publication, UN, 2022).

19. J. M. Aburto, J. Schöley, I. Kashnitsky, L. Zhang, C. Rahal, T. I. Missov, M. C. Mills, J. B. Dowd, R. Kashyap , Quantifying impacts of the COVID-19 pandemic through life-expectancy losses: A population-level study of 29 countries. *Int. J. Epidemiol.* **51**, 63–74 (2022).

20. P. Geldsetzer, T. Mukama, N. K. Jawad, T. Riffe, A. Rogers, N. Sudharsanan , Sex differences in the mortality rate for coronavirus disease 2019 compared to other causes of death: An analysis of population-wide data from 63 countries. *Eur. J. Epidemiol.* **37**, 797–806 (2022).

21. B. R. Ambedkar, *Annihilation of Caste with a Reply to Mahatma Gandhi* (1945).

22. V. Xaxa, Tribes as indigenous people of India. *Econ. Political Wkly.* **34**, 3589–3595 (1999).

23. Sachar Committee, Government of India, Social, Economic and Educational Status of the Muslim Community of India, *Prime Minister's High Level Committee, Cabinet Secretariat, Government of India* (2006).

24. S. Vyas, P. Hathi, A. Gupta, Social disadvantage, economic inequality, and life expectancy in nine Indian states. *Proc. Natl. Acad. Sci. U.S.A.* **119**, e2109226119 (2022).

25. N. Goldman, T. Andrasfay, Life expectancy loss among Native Americans during the COVID-19 pandemic. *Demogr. Res.* **47**, 233–246 (2022).

26. M. Banaji, A. Gupta, Estimates of pandemic excess mortality in India based on civil registration data. *PLoS Glob. Public Health* **2**, e0000803 (2022).

27. covid19india.org, COVID-19 India - covid19india.org (2023). Accessed on April 2023.

28. V. Knutson, S. Aleshin-Guendel, A. Karlinsky, W. Msemburi, J. Wakefield, Estimating global and country-specific excess mortality during the COVID-19 pandemic. arXiv:2205.09081 [stat.AP] (2022).

29. F. B. Ahmad, J. A. Cisewski, A. Miniño, R. N. Anderson, Provisional mortality data – United States, 2020. *Morb. Mortal. Wkly. Rep.* **70**, 519–522 (2021).

30. P. Jha, Y. Deshmukh, C. Tumbe, W. Suraweera, A. Bhowmick, S. Sharma, P. Novosad, S. H. Fu, L. Newcombe, H. Gelband, P. Brown , COVID mortality in India: National survey data and health facility deaths. *Science* **375**, 667–671 (2022).

31. A. Anand, J. Sandefur, A. Subramanian, “Three New Estimates of India’s all-Cause Excess Mortality during the COVID-19 Pandemic,” (Tech. Rep. Center for Global Development, 2021).

32. Y. Mo, Q. Feng, D. Gu, Impacts of the COVID-19 pandemic on life expectancy at birth in Asia. *BMC Public Health* **23**, 1508 (2023).

33. A. Somanchi, Missing the poor, big time: A critical assessment of the consumer pyramids household survey, SocArXiv [Preprint] (2021); https://doi.org/10.31235/osf.io/qmce9.

34. A. Malani, S. Ramachandran, Using household rosters from survey data to estimate all-cause excess death rates during the COVID pandemic in India. *J. Dev. Econ.* **159**, 102988 (2022).

35. J. M. Aburto, J. Schöley, I. Kashnitsky, R. Kashyap, Life expectancy declines in Russia during the COVID-19 pandemic in 2020. *Int. J. Epidemiol.* **51**, 1695–1697 (2022).

36. R. Kashyap, Is prenatal sex selection associated with lower female child mortality? *Popul. Stud. (Camb.)* **73**, 57–78 (2019).

37. C. Z. Guilmoto, N. Saikia, V. Tamrakar, J. K. Bora, Excess under-5 female mortality across India: A spatial analysis using 2011 census data. *Lancet Glob. Health* **6**, e650–e658 (2018).

38. A. Maharatna, Fertility, mortality and gender bias among tribal population: An Indian perspective. *Soc. Sci. Med.* **50**, 1333–1351 (2000).

39. N. Saikia, J. K. Bora, Gender difference in health-care expenditure: Evidence from India human development survey. *PLOS ONE* **11**, e0158332 (2016).

40. P. Dupas, R. Jain, “Women left behind: Gender disparities in utilization of government health insurance in India” (Tech. rep., National Bureau of Economic Research, 2021).

41. R. Jain, P. Dupas, The effects of India’s COVID-19 lockdown on critical non-COVID health care and outcomes: Evidence from dialysis patients. *Soc. Sci. Med.* **296**, 114762 (2022).

42. G. Thejesh, K. Ranaware, K. Sharma, Aman, Of denial and data deaths due to India’s covid-19 national lockdown. *Econ. Political Wkly.* **56**, 37–41 (2021).

43. M. V. Murhekar, T. Bhatnagar, J. W. V. Thangaraj, V. Saravanakumar, M. S. Kumar, S. Selvaraju, K. Rade, C. P. G. Kumar, R. Sabarinathan, A. Turuk, S. Asthana, R. Balachandar, S. D. Bangar, A. K. Bansal, V. Chopra, D. das, A. K. Deb, K. R. Devi, V. Dhikav, G. R. Dwivedi, S. M. S. Khan, M. S. Kumar, A. Laxmaiah, M. Madhukar, A. Mahapatra, C. Rangaraju, J. Turuk, R. Yadav, R. Andhalkar, K. Arunraj, D. K. Bharadwaj, P. Bharti, D. Bhattacharya, J. Bhat, A. S. Chahal, D. Chakraborty, A. Chaudhury, H. Deval, S. Dhatrak, R. Dayal, D. Elantamilan, P. Giridharan, I. Haq, R. K. Hudda, B. Jagjeevan, A. Kalliath, S. Kanungo, N. N. Krishnan, J. S. Kshatri, A. Kumar, N. Kumar, V. G. V. Kumar, G. G. J. N. Lakshmi, G. Mehta, N. K. Mishra, A. Mitra, K. Nagbhushanam, A. Nimmathota, A. R. Nirmala, A. K. Pandey, G. V. Prasad, M. A. Qurieshi, S. D. Reddy, A. Robinson, S. Sahay, R. Saxena, K. Sekar, V. K. Shukla, H. B. Singh, P. K. Singh, P. Singh, R. Singh, N. Srinivasan, D. S. Varma, A. Viramgami, V. C. Wilson, S. Yadav, S. Yadav, K. Zaman, A. Chakrabarti, A. das, R. S. Dhaliwal, S. Dutta, R. Kant, A. M. Khan, K. Narain, S. Narasimhaiah, C. Padmapriyadarshini, K. Pandey, S. Pati, S. Patil, H. Rajkumar, T. Ramarao, Y. K. Sharma, S. Singh, S. Panda, D. C. S. Reddy, B. Bhargava, T. Anand, G. R. Babu, H. Chauhan, T. Dikid, R. R. Gangakhedkar, S. Kant, S. Kulkarni, J. P. Muliyil, R. M. Pandey, S. Sarkar, N. Shah, A. Shrivastava, S. K. Singh, S. Zodpe, A. Hindupur, P. R. Asish, M. Chellakumar, D. Chokkalingam, S. Dasgupta, M. M. E. Gowtham, A. Jose, K. Kalaiyarasi, N. N. Karthik, T. Karunakaran, G. Kiruthika, H. Dinesh Kumar, S. Sarath Kumar, M. P. Sarath Kumar, E. Michaelraj, J. Pradhan, E. B. Arun Prasath, D. Gladys Angelin Rachel, S. Rani, A. Rozario, R. Sivakumar, P. Gnana Soundari, K. Sujeetha, A. Vinod , SARS-CoV-2 seroprevalence among the general population and healthcare workers in India, December 2020–January 2021. *Int. J. Infect. Dis.* **108**, 145–155 (2021).

44. M. O'Driscoll, G. Ribeiro dos Santos, L. Wang, D. A. T. Cummings, A. S. Azman, J. Paireau, A. Fontanet, S. Cauchemez, H. Salje, Age-specific mortality and immunity patterns of SARS-CoV-2. *Nature* **590**, 140–145 (2021).

45. A. Kurtz, K. Grant, R. Marano, A. Arrieta, K. Grant Jr, W. Feaster, C. Steele, L. Ehwerhemuepha, Long-term effects of malnutrition on severity of COVID-19. *Sci. Rep.* **11**, 14974 (2021).

46. A. S. Shonchoy, S. Dhongde, E. Asker, COVID-19 lockdown and neonatal mortality: Evidence from India. (2023); https://ssrn.com/abstract=4518698.

47. Z. Wagner, S. Heft-Neal, Z. Wang, R. Jing, E. Bendavid, Infant and neonatal mortality during the covid-19 pandemic: An interrupted time series analysis from five low-and middle-income countries. medRxiv [Preprint] (2023). https://doi.org/10.1101/2023.08.03.23293619.

48. J. Drèze, A. Somanchi, The Covid-19 crisis and people's right to food, SocArXiv [Preprint] (2021); https://doi.org/10.31235/osf.io/ybrmg.

49. A. Summan, A. Nandi, A. Shet, R. Laxminarayan, The effect of the COVID-19 pandemic on routine childhood immunization coverage and timeliness in India: Retrospective analysis of the National Family Health Survey of 2019–2021 data. *Lancet Reg. Health–Southeast Asia* **8**, 100099 (2023).

50. M. Pai, T. Kasaeva, S. Swaminathan, Covid-19's devastating effect on tuberculosis care–A path to recovery. *N. Engl. J. Med.* **386**, 1490–1493 (2022).

51. V. Kumari, K. Mehta, R. Choudhary, COVID-19 outbreak and decreased hospitalisation of pregnant women in labour. *Lancet Glob. Health* **8**, e1116–e1117 (2020).

52. A. T. Levin, N. Owusu-Boaitey, S. Pugh, B. K. Fosdick, A. B. Zwi, A. Malani, S. Soman, L. Besançon, I. Kashnitsky, S. Ganesh, A. McLaughlin, G. Song, R. Uhm, D. Herrera-Esposito, G. de los Campos, A. C. Peçanha Antonio, E. B. Tadese, G. Meyerowitz-Katz, Assessing the burden of COVID-19 in developing countries: Systematic review, meta-analysis and public policy implications. *BMJ Glob. Health* **7**, e008477 (2022).

53. S. Yasir, India is scapegoating Muslims for the spread of the Coronavirus. *Foreign Policy* **22** (2020). https://foreignpolicy.com/2020/04/22/india-muslims-coronavirus-scapegoat-modi-hindu-nationalism/

54. T. Andrasfay, N. Goldman, Reductions in US life expectancy during the COVID-19 pandemic by race and ethnicity: Is 2021 a repetition of 2020? *PLOS ONE* **17**, e0272973 (2022).

55. E. Arias, J. Xu, United States life tables, 2019. *Natl. Vital Stat. Rep.* **70**, 1–59 (2022).

56. E. Arias, J. Xu, United States life tables, 2020. *Natl. Vital Stat. Rep.* **71**, 1–18 (2022).

57. M. R. Nepomuceno, I. Klimkin, D. A. Jdanov, A. Alustiza-Galarza, V. M. Shkolnikov, Sensitivity analysis of excess mortality due to the COVID-19 pandemic. *Popul. Dev. Rev.* **48**, 279–302 (2022).

58. V. M. Shkolnikov, I. Klimkin, M. McKee, D. A. Jdanov, A. Alustiza-Galarza, L. Németh, S. A. Timonin, M. R. Nepomuceno, E. M. Andreev, D. A. Leon, What should be the baseline when calculating excess mortality? New approaches suggest that we have underestimated the impact of the COVID-19 pandemic and previous winter peaks. *SSM-Popul. Health* **18**, 101–118 (2022).

59. M. Banaji, "What lessons do India's Covid-19 serosurveys hold?" *Scroll.in*, 06 February 2021.

60. M. V. Murhekar, T. Bhatnagar, S. Selvaraju, V. Saravanakumar, J. W. V. Thangaraj, N. Shah, M. S. Kumar, K. Rade, R. Sabarinathan, S. Asthana, R. Balachandar, S. D. Bangar, A. K. Bansal, J. Bhat, V. Chopra, D. das, A. K. Deb, K. R. Devi, G. R. Dwivedi, S. M. S. Khan, C. P. G. Kumar, M. S. Kumar, A. Laxmaiah, M. Madhukar, A. Mahapatra, S. S. Mohanty, C. Rangaraju, A. Turuk, D. K. Baradwaj, A. S. Chahal, F. Debnath, I. Haq, A. Kalliath, S. Kanungo, J. S. Kshatri, G. G. J. N. Lakshmi, A. Mitra, A. R. Nirmala, G. V. Prasad, M. A. Qurieshi, S. Sahay, R. K. Sangwan, K. Sekar, V. K. Shukla, P. K. Singh, P. Singh, R. Singh, D. S. Varma, A. Viramgami, S. Panda, D. C. S. Reddy, B. Bhargava, R. Andhalkar, A. Chaudhury, H. Deval, S. Dhatrak, R. R. Gupta, E. Ilayaperumal, B. Jagjeevan, R. C. Jha, K. Kiran, N. N. Krishnan, A. Kumar, V.G. V. Kumar, K. Nagbhushanam, A. Nimmathota, A. K. Pandey, H. S. Pawar, K. S. Rathore, A. Robinson, H. B. Singh, V. C. Wilson, A. Yadav, R. Yadav, T. Karunakaran, J. Pradhan, T. Sivakumar, A. Jose, K. Kalaiyarasi, S. Dasgupta, R. Anusha, T. Anand, G. R. Babu, H. Chauhan, T. Dikid, R. R. Gangakhedkar, S. Kant, S. Kulkarni, J. P. Muliyil, R. M. Pandey, S. Sarkar, A. Shrivastava, S. K. Singh, S. Zodpey, A. das, P. das, S. Dutta, R. Kant, K. Narain, S. Narasimhaiah, S. Pati, S. Patil, H. Rajkumar, T. Ramarao, K. Sarkar, S. Singh, G. S. Toteja, K.

Zaman, SARS-CoV-2 antibody seroprevalence in India, August–September, 2020: Findings from the second nationwide household serosurvey. *Lancet Glob. Health* **9**, e257–e266 (2021).

61. Registrar General and Census Commissioner of India, *Compendium of India's Fertility and Mortality Indicators, 1971–2013* (Government of India, 2013).

62. M. C. Castro, S. Gurzenda, C. M. Turra, S. Kim, T. Andrasfay, N. Goldman, Reduction in life expectancy in Brazil after COVID-19. *Nat. Med.* **27**, 1629–1635 (2021).

63. F. Fernandes, C. M. Turra, G. V. França, M. C. Castro, Mortality by cause of death in Brazil: Effects of the COVID-19 pandemic and contribution to changes in life expectancy at birth. medRxiv 2023.02.13.23285842 [Preprint] (2023).

64. V. M. García-Guerrero, H. Beltrán-Sánchez, Heterogeneity in excess mortality and its impact on loss of life expectancy due to COVID-19: Evidence from Mexico. *Can. Stud. Popul.* **48**, 165–200 (2021).

65. A. Rangachev, G. K. Marinov, M. Mladenov, The demographic and geographic impact of the COVID pandemic in Bulgaria and Eastern Europe in 2020. *Sci. Rep.* **12**, 6333 (2022).

66. N. Saikia, K. Kumar, B. Das, Death registration coverage 2019–2021, India. *Bull. World Health Organ.* **101**, 102–110 (2023).

67. L. V. Zimmermann, M. Salvatore, G. R. Babu, B. Mukherjee, Estimating COVID-19–related mortality in India: An epidemiological challenge with insufficient data. *Am. J. Public Health* **111**, S59–S62 (2021).

68. S. KC, M. Dhakad, Impact of the COVID-19 pandemic on the age-sex pattern of COVID-19 deaths in India. *Asian Popul. Stud.* **19**, 148–167 (2023).

69. V. Adjiwanou, N. Alam, L. Alkema, G. Asiki, A. Bawah, D. Béguy, V. Cetorelli, A. Dube, D. Feehan, A. B. Fisker, Measuring excess mortality during the COVID-19 pandemic in low-and lower-middle income countries: The need for mobile phone surveys. SocArXiv (2020); https://doi.org/10.31235/osf.io/4bu3q.

70. I. M. Timaeus, Measurement of adult mortality in less developed countries: A comparative review. *Popul. Index* **57**, 552–568 (1991).

71. S.-E. Mamelund, J. Dimka, Not the great equalizers: Covid-19, 1918–20 influenza, and the need for a paradigm shift in pandemic preparedness. *Popul. Stud.* **75**, 179–199 (2021).

72. I. Klein, Death in India, 1871–1921. *J. Asian Stud.* **32**, 639–659 (1973).

73. B. G. Link, J. Phelan, Social conditions as fundamental causes of disease. *J. Health Soc. Behav.* **35**, 80–94 (1995).

74. International Institute for Population Sciences - IIPS/India and ICF., *India National Family Health Survey NFHS-5 2019–21* (Mumbai, India: IIPS and ICF, 2022).

75. Registrar General of Census Commissioner of India, *Vital Statistics of India: 2020* (2022).

76. T. A. Moultrie, R. Dorrington, A. Hill, K. Hill, I. Timæus, B. Zaba, *Tools for Demographic Estimation* (International Union for the Scientific Study of Population, 2013).

77. A. Gupta, Seasonal variation in infant mortality in india. *Popul. Stud. (Camb.)* **76**, 535–552 (2022).

78. S. Preston, P. Heuveline, M. Guillot, *Demography: Measuring and Modeling Population Processes* (Wiley-Blackwell Publishing, 2001).

79. E. E. Arriaga, Measuring and explaining the change in life expectancies. *Demography* **21**, 83–96 (1984).

80. A. C. Cameron, D. L. Miller, A practitioner's guide to cluster-robust inference. *J. Hum. Resour.* **50**, 317–372 (2015).

81. D. C. Brown, J. T. Lariscy, L. Kalousová, Comparability of mortality estimates from social surveys and vital statistics data in the United States. *Popul. Res. Policy Rev.* **38**, 371–401 (2019).

82. J. R. Warren, C. Milesi, K. Grigorian, M. Humphries, C. Muller, E. Grodsky, Do inferences about mortality rates and disparities vary by source of mortality information? *Ann. Epidemiol.* **27**, 121–127 (2017).

83. N. Franz, A. Gupta, D. Spears, D. Coffey, Uncertainty about maternal mortality in India: New, higher estimates from the National Family Health Survey-4. SocArXiv (2022); https://doi.org/10.31235/osf.io/8as72.

84. "National Commission on Population, Population projections for India and States 2011–2036: Report of the Technical Group on Population Projections Constituted by the National Commission on Population" (Tech. Rep., Government of India, 2019).
